# Zyxin-Siah2–Lats2 axis mediates cooperation between Hippo and TGF-β signalling pathways

**DOI:** 10.1038/ncomms11123

**Published:** 2016-03-31

**Authors:** Biao Ma, Hongcheng Cheng, Ruize Gao, Chenglong Mu, Ling Chen, Shian Wu, Quan Chen, Yushan Zhu

**Affiliations:** 1 State Key Laboratory of Medicinal Chemical Biology, Tianjin Key Laboratory of Protein Sciences, College of Life Sciences, Nankai University, Tianjin 300071, China; 2 State Key Laboratory of Membrane Biology, Institute of Zoology, Chinese Academy of Sciences, Beijing 100101, China

## Abstract

The evolutionarily conserved Hippo pathway is a regulator that controls organ size, cell growth and tissue homeostasis. Upstream signals of the Hippo pathway have been widely studied, but how microenvironmental factors coordinately regulate this pathway remains unclear. In this study, we identify LIM domain protein Zyxin, as a scaffold protein, that in response to hypoxia and TGF-β stimuli, forms a ternary complex with Lats2 and Siah2 and stabilizes their interaction. This interaction facilitates Lats2 ubiquitination and degradation, Yap dephosphorylation and subsequently activation. We show that Zyxin is required for TGF-β and hypoxia-induced Lats2 downregulation and deactivation of Hippo signalling in MDA-MB-231 cells. Depletion of Zyxin impairs the capability of cell migration, proliferation and tumourigenesis in a xenograft model. Zyxin is upregulated in human breast cancer and positively correlates with histological stages and metastasis. Our study demonstrates that Zyxin-Lats2–Siah2 axis may serve as a potential therapeutic target in cancer treatment.

## Abstract (web summary)

Hippo and TGF-β are crucial signalling pathways involved in the development of various types of tumours. Here, the authors demonstrate that TGF-β can directly regulate Hippo pathway through the stabilization of the scaffold protein Zyxin, which forms a ternary complex with Siah2 and Lats2 promoting Lats2 degradation and YAP activation.

## Graphical abstract

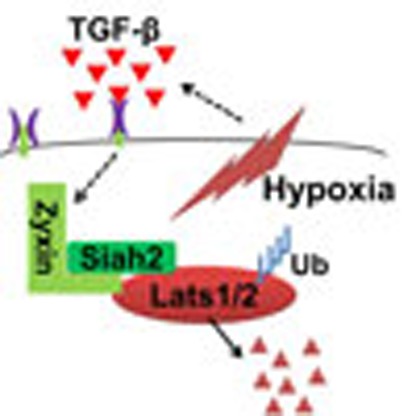

The Hippo signalling pathway has been implicated in controlling the size of developing organs by coordinated regulation of cell growth, proliferation and apoptosis^1^,^2^,^3^,^4^. Dysregulation of the Hippo pathway contributes to loss of cell contact inhibition, cell over-growth and epithelial–mesenchymal transition (EMT)^5^,^6^. All these factors can promote tumourigenesis and metastasis. The large tumour suppressors (Lats1/2), the homologues of drosophila Warts, are serine/ threonine kinases and the core components of the Hippo pathway^7^,^8^,^9^,^10^. When Hippo signalling is activated, Lats1/2 are phosphorylated and activated by Mst1/2, which allows Lats1/2 to phosphorylate the oncoproteins Yap and Taz (ref. 11), initiating their cytoplasm retention and deactivation, resulting in inhibition of cell growth and tumourigenesis^5^,^12^. Reduced expression of Lats has been observed in a variety of human cancers such as colorectal cancers, prostate cancers and breast cancers^13^,^14^,^15^,^16^, which reveals that Lats downregulation may contribute to tumourigenesis.

Upstream signals that regulate the Hippo pathway were widely studied, including physical stimuli from cell–cell contact^5^, hypoxia^15^,^17^ and diffused chemical signal from growth factors^18^. Transforming growth factor beta (TGF-β) was one of these growth factors that previously reported involved in regulation of the Hippo signalling^19^,^20^, but the molecular mechanism remained elusive. We previously reported that hypoxia could induce Lats2 degradation through activating E3 ubiquitin ligase Siah2 (ref. 15), this is step one for hypoxic tumour cells to shut down the Hippo signalling. In this work, we present step two by showing that under pathophysiological conditions, such as hypoxia microenvironment, not only the activity of Siah2 is elevated, but also the secretion of TGF-β, a growth factor that has complicated function in tumour development. We show that LIM domain protein Zyxin, as a scaffold protein, in response to hypoxia and TGF-β stimuli, forms a ternary complex with Siah2 and Lats2, thus stabilizes their interaction, and facilitates deactivation of the Hippo signalling, thereby promoting tumour progression. We suggest that hypoxia-induced autocrine of TGF-β may serve as a magnifying mechanism that works together with Siah2 to negatively regulate Hippo signalling. Thus, our findings provide insights into the mechanism of TGF-β-induced deactivation of the Hippo pathway and a potential role of this regulation in tumour progression.

## Results

### Hypoxia-induced TGF-β activates Yap through Zyxin

**Figure 1:**
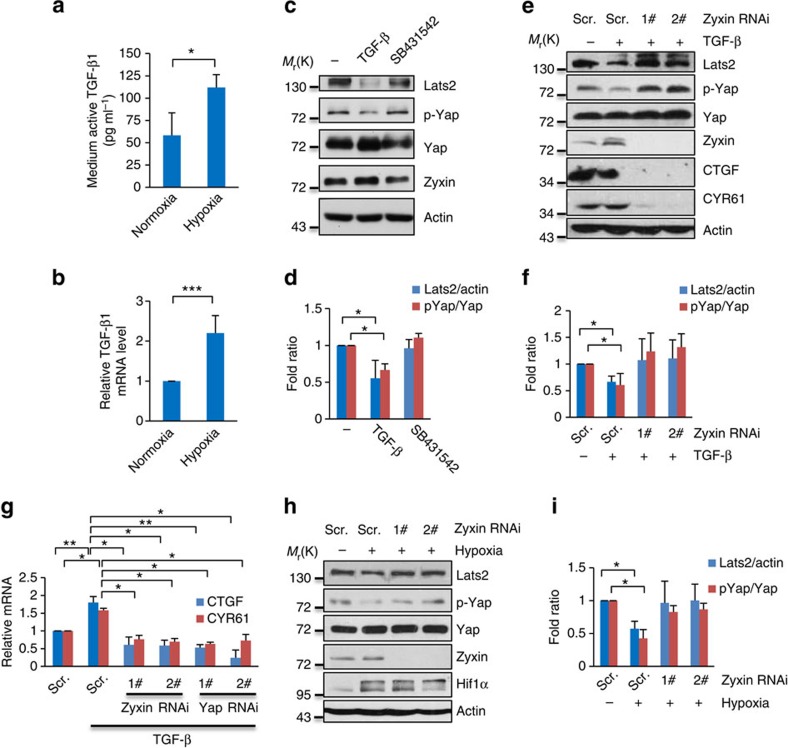
Hypoxia-induced TGF-β secretion contributes to deactivation of Hippo signalling through Zyxin. (**a**) MDA-MB-231 cells were cultured in serum-free DMEM under normoxic or hypoxic conditions for 12 h. Culture mediums were collected and active TGF-β1 was measured by Elisa. Student's *t*-test was applied. (**b**) MDA-MB-231 cells were cultured under normoxia or hypoxia for 12 h. *TGF-β*1 mRNA level was analysed by real-time PCR (RT-qPCR). Student's *t*-test was applied. (**c**,**d**) MDA-MB-231 cells were treated with 5 ng ml^−1^ TGF-β or 5 μm ml^−1^ SB431542 for 12 h, and then collected for western blotting using the indicated antibodies (**c**) and quantification of Lats2 (normalized to actin) and p-Yap (normalized to Yap) protein levels (**d**). Ratio *t*-test was applied. (**e**,**f**) Scramble and Zyxin-knockdown MDA-MB-231 cells were treated with 5 ng ml^−1^ TGF-β for 12 h, and then collected for western blotting using the indicated antibodies (**e**) and quantification of Lats2 (normalized to actin) and p-Yap (normalized to Yap) protein levels (**f**). Ratio *t*-test was applied. (**g**) Zyxin-knockdown and Yap-knockdown MDA-MB-231 cells were treated with 5 ng ml^−1^ TGF-β for 12 h. Total RNA was extracted and subjected to RT-qPCR analysis for the indicated genes. Student's *t*-test was applied. (**h**,**i**) Scramble and Zyxin-knockdown MDA-MB-231 cells were cultured under normoxia or hypoxia for 6 h, and then collected for western blotting using the indicated antibodies (**h**) and quantification of Lats2 (normalized to actin) and p-Yap (normalized to Yap) protein levels (**i**). Ratio *t*-test was applied. All data are mean of *n*=3 independent experiments. All error bars indicate s.d. *P<0.05; **P<0.01; ***P<0.001.

Hypoxia is an important microenvironment factor that contributes to tumour progression and it is also a common feature in solid tumours^21^. Hypoxic tumour cells can secret variety of cytokines to sustain their survival under hypoxia^22^ and autocrine TGF-β is necessary for the growth and survival of human breast cancer MDA-MB-231 cells^23^. So we wondered whether hypoxic condition could induce the secretion of TGF-β. Indeed, hypoxic conditions not only dramatically increased the level of autocrine of TGF-β in MDA-MB-231 cells (Fig. 1a), but also increased the transcriptional level of TGF-β (Fig.1b). Based on our previous finding that hypoxia could deactivate the Hippo signalling^15^, thus giving us a notion that hypoxia-induced secretion of TGF-β may also contributes to deactivation of the Hippo signalling since TGF-β was reported to facilitating the deactivation of the Hippo signalling in an unknown mechanism^19^,^20^. Intriguingly, we found that Zyxin, a scaffold protein that induced by TGF-β (ref. 24) (Supplementary Fig. 1a,b), involved in TGF-β induced deactivation of the Hippo signalling (Fig. 1c,d). Efficient knockdown of Zyxin almost completely attenuated TGF-β-induced downregulation of both Lats2 and p-Yap (Fig. 1e,f). Furthermore, the Hippo target genes *CTGF* and *CYR61* were also downregulated in both protein and transcriptional levels in Zyxin-knockdown cells (Fig. 1e,g), which have a similar pattern of Yap knockdown (Fig. 1g). Consistently, knockdown of Zyxin attenuated hypoxia-induced Lats2 degradation and Yap dephosphorylation (Fig. 1h,i), suggesting that Zyxin is required for hypoxia and TGF-β-induced deactivation of the Hippo signalling, which means hypoxia-induced TGF-β may also participate in regulation of the Hippo signalling through Zyxin.

### Loss of Zyxin stabilizes Lats2 and activates Hippo signalling

**Figure 2:**
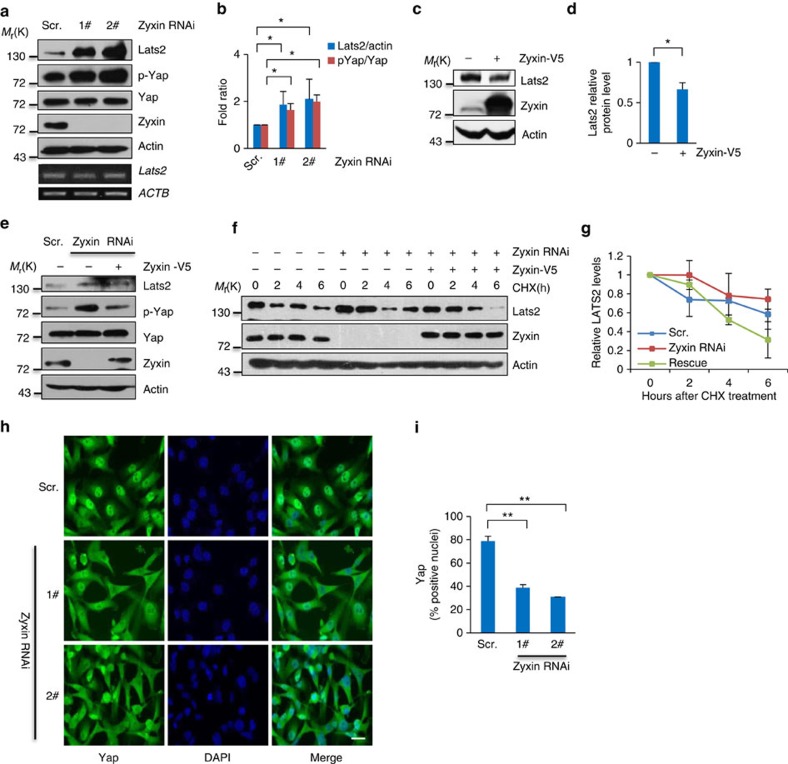
Loss of Zyxin stabilizes Lats2 and activates the Hippo signalling. (**a**,**b**) Cells stably expressed short hairpin RNA (shRNA) against Zyxin were collected for immunoblotting with indicated antibodies or by PCR with reverse transcription (RT–PCR) for the indicated genes (**a**) and quantification of Lats2 (normalized to actin) and p-Yap (normalized to Yap) protein levels (**b**). Ratio *t*-test was applied. (**c**,**d**) Cells transiently transfected with Zyxin for 24 h then were collected for immunoblotting with the indicated antibodies (**c**) and quantification of Lats2 (normalized to actin) protein levels (**d**). Ratio *t*-test was applied. (**e**) Zyxin-knockdown cells were transfected with wild-type Zyxin. Twenty-four hours later, cells were collected for immunoblotting with indicated antibodies. (**f**) Cells were transfected with Zyxin, treated with 100 μg ml^−1^ cycloheximide (CHX), collected at indicated the time points and then immunoblotted with the indicated antibodies. (**g**) Quantification of Lats2 protein levels (normalized to actin). (**h**) Zyxin-knockdown MDA-MB-231 cells were stained with 4,6-diamidino-2-phenylindole (DAPI) and antibody against Yap. Scale bars, 25 μm. (**i**) Quantitative analysis of Yap nuclear translocation. Student's *t*-test was applied. All data are mean of *n*=3 independent experiments. All error bars indicate s.d. *P<0.05; **P<0.01.

Next, we want to further investigate the mechanism how Zyxin affects the Hippo signalling. We found that knockdown of Zyxin could stabilize Lats2 in both MDA-MB-231 and HeLa cells (Fig. 2a,b and Supplementary Fig. 1c,d), and these alterations were not caused by upregulation of Lats2 messenger RNA (mRNA) levels (Fig. 2a), indicating that Zyxin may function as an endogenous regulator of Lats2 at posttranslational level. Ectopic expression of Zyxin could induce the downregulation of both endogenous and ectopic expressed Lats2 (Fig. 2c,d and Supplementary Fig. 2b), which is consistent with previous loss of function data. Re-expression of Zyxin in Zyxin-knockdown cells attenuated the effect of accumulation of Lats2 and phosphor-Yap (Fig. 2e), besides, the half-life of Lats2 was also rescued (Fig. 2f,g), indicating that the accumulation of Lats2 and phosphor-Yap is not due to an off-target effect.

Yap phosphorylation on Serine 127 is a direct readout of Lats kinase activity. Concomitant with Zyxin depletion induced stabilization of Lats2; Yap-S127 phosphorylation levels were significantly enhanced in both MDA-MB-231 and HeLa cells (Fig. 2a,b and Supplementary Fig. 1c,d). Conversely, ectopic expression of Zyxin promoted reduction of Yap-S127 phosphorylation (Fig. 2e) and increased Yap nuclear translocation (Supplementary Fig. 1e), whereas knockdown of Zyxin resulted in decreased Yap nuclear localization (Fig. 2h,i). Taken together, these results indicate that Zyxin may function as a negative regulator of Hippo signalling through controlling Lats2 stability and Yap activity.

### Zyxin facilitates Siah2-induced Lats2 destabilization

**Figure 3:**
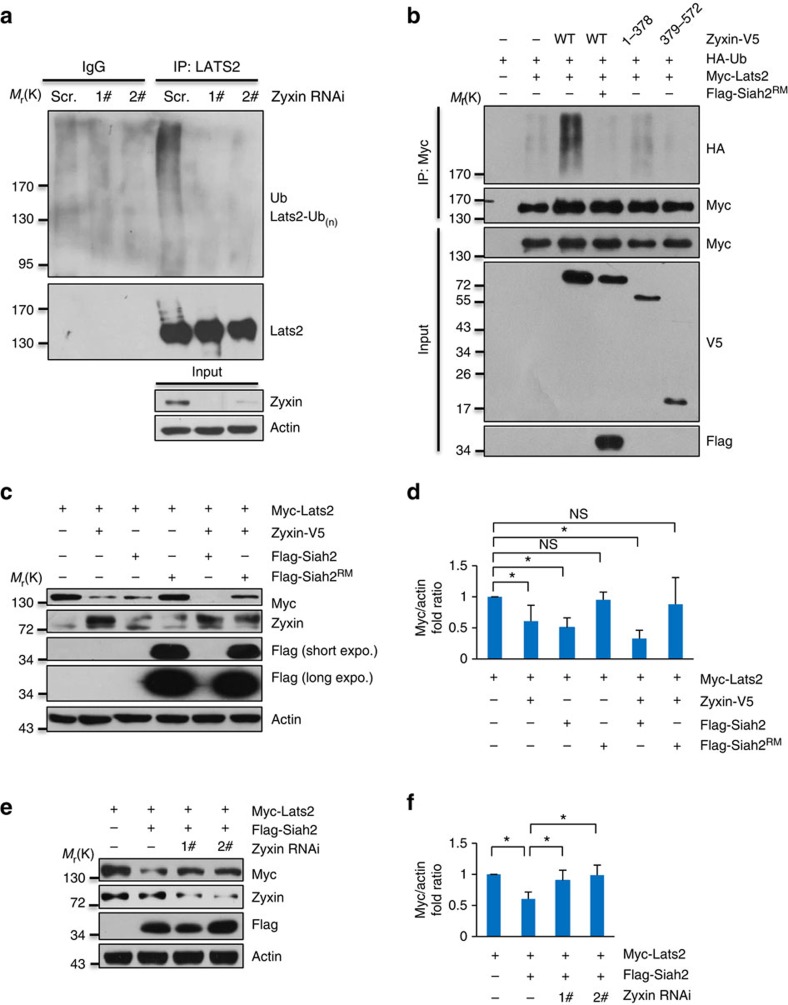
Zyxin facilitates Lats2 ubiquitination and degradation. (**a**) Decreased Lats2 ubiquitylation level by Zyxin-knockdown *in vivo*. (**b**) Full-length Zyxin-induced Lats2 ubiquitylation was attenuated by ectopic expression of Siah2^RM^. (**c**,**d**) Zyxin-enhanced Siah2-induced Lats2 degradation (**c**) and quantification of Myc-Lats2 (normalized to actin) protein levels (**d**). Ratio *t*-test was applied. (**e**,**f**) Zyxin was required for Siah2-induced Lats2 degradation (**e**) and quantification of Myc-Lats2 (normalized to actin) protein levels (**f**). Ratio *t*-test was applied. All data are mean of *n*=3 independent experiments. All error bars indicate s.d. *P<0.05; NS as not significant.

We and others have reported that Lats2 destabilization is mainly regulated by ubiquitin proteasome pathway^15^,^17^,^25^. So we first analysed Lats2 ubiquitination levels in Zyxin knockdown MDA-MB-231 cells. As expected, knockdown of Zyxin attenuated Lats2 ubiquitination (Fig. 3a), indicating that Zyxin may regulate Lats2 stability through promoting its polyubiquitination. Consistently, ectopic expression of full length of Zyxin indeed promoted Lats2 ubiquitination, whereas the Zyxin truncation mutants Zyxin (1-378) and Zyxin (379-572) had no such effect (Fig. 3b), indicating that both N-terminal and C-terminal of Zyxin are both required for its effect on Lats2 ubiquitination. Intriguingly, Zyxin-induced Lats2 unbiquitination was completely abolished by a dominant negative mutant of E3 ligase Siah2 (Siah2^RM^)^15^ (Fig. 3b), indicating that Zyxin may promote Lats2 ubiquitination through Siah2. Strikingly, co-expression of Siah2 and Zyxin efficiently destabilized ectopic expressed Lats2, whereas Siah2^RM^ could not (Fig. 3c,d). These results thus provide us an insight that Zyxin may together with Siah2 to facilitate Lats2 degradation. Indeed, knockdown of Zyxin inhibited Siah2-induced Lats2 destabilization (Fig. 3e,f), indicating that Zyxin is at least partially required for Siah2-induced Lats2 turnover. This is consistent with our previous data that depletion of Zyxin compromises Siah2-induced Lats2 degradation under hypoxic conditions (Fig. 1h,i), suggesting that a possibility of Zyxin may be a part of Siah2–Lats2 degradation complex.

### Zyxin forms a ternary complex with Siah2 and Lats2

**Figure 4:**
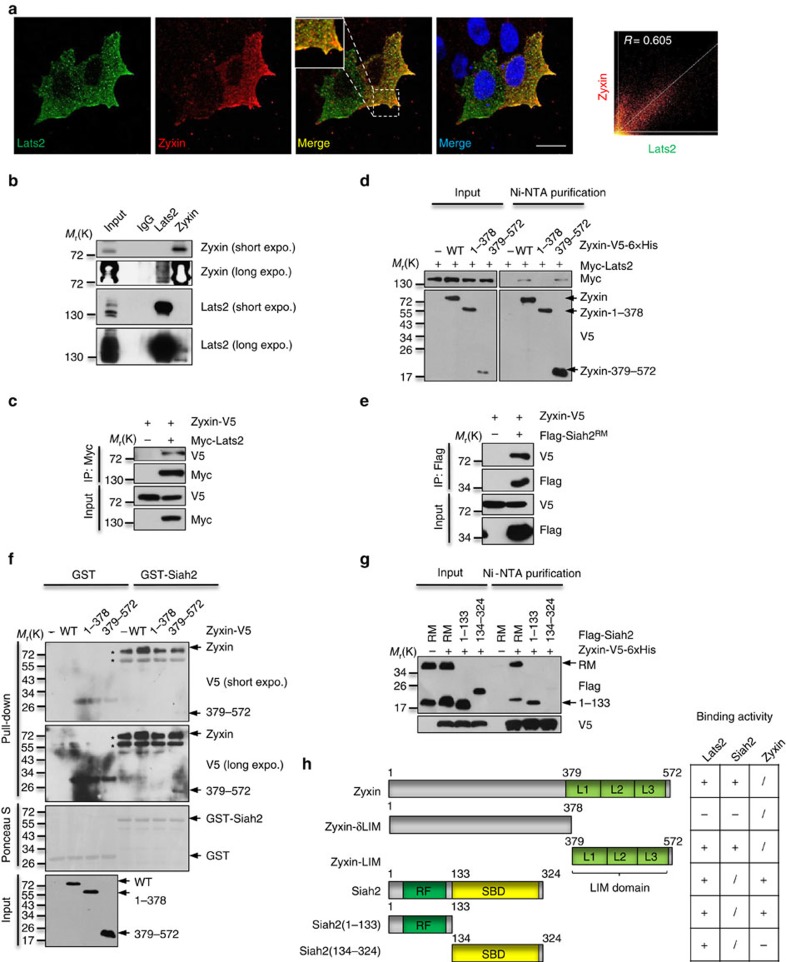
Zyxin interacts with Siah2 and Lats2. (**a**) HeLa cells were transiently transfected with Myc-Lats2 and Zyxin-V5 and subjected to immunostaining with anti-Myc and anti-V5 antibodies. Images were collected by confocal microscopy. *R* is the colocalization correlation coefficient. Scale bars, 20 μm. (**b**) Endogenous interaction between Lats2 and Zyxin in MDA-MB-231 cells was analysed by immunoprecipitation. (**c**) Coimmunoprecipitation of exogenously expressed Myc-Lats2 with Zyxin-V5. (**d**) Dissection of the critical regions of Zyxin binding to Lats2 in HEK293T cells. (**e**) Coimmunoprecipitation of exogenously expressed Flag-Siah2^RM^ with Zyxin-V5. (**f**) Dissection of the critical regions of Zyxin binding to Siah2 through GST pull-down. Asterisk indicates non-specific binding. (**g**) Dissection of the critical regions of Siah2 binding to Zyxin in HEK293T cells. (**h**) Schematic drawing of critical regions of Siah2 and Zyxin and their responses to interaction with Lats2. ‘+' indicates as binding, ‘−' as no binding, and ‘/' as not examined. L, lim domain; RF, ring finger domain; SBD, substrate binding domain.

**Figure 5:**
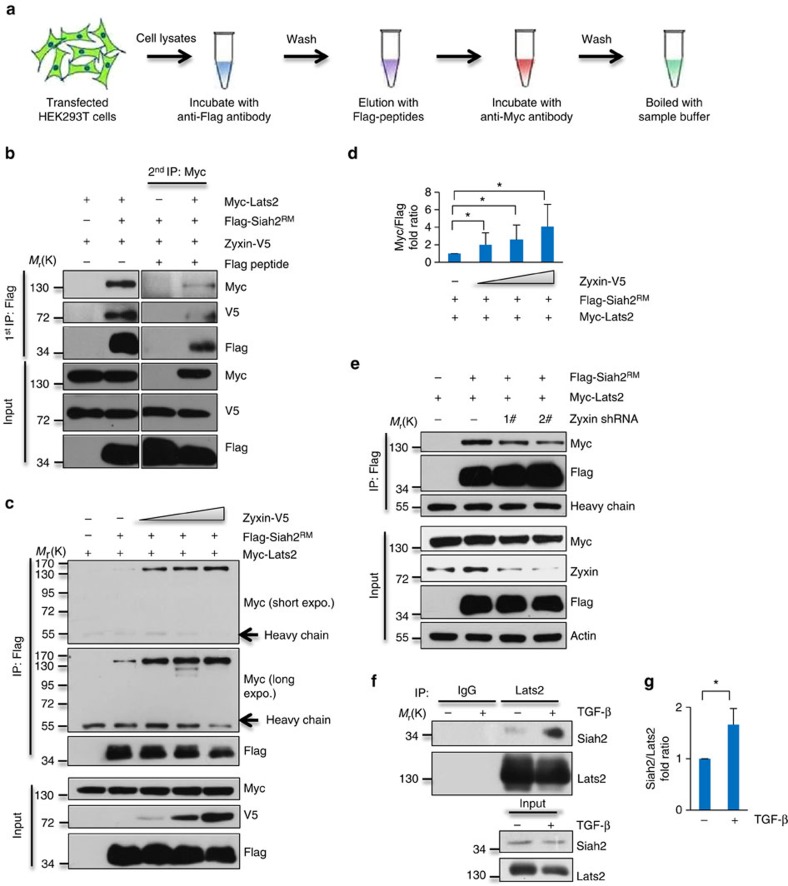
Zyxin forms a ternary complex with Siah2 and Lats2 and enhances Siah2–Lats2 binding. (**a**,**b**) Double coimmunoprecipitation was performed as depicted (**a**) and the interactions were determined by western blotting (**b**). (**c**,**d**) Enhanced interaction between exogenously expressed Myc-Lats2 with Flag-Siah2^RM^ in the presence of increased amount of ectopic expression of Zyxin (**c**) and quantification of Myc-Lats2 (normalized to Flag-Siah2^RM^) protein levels (**d**). Ratio t-test was applied. (**e**) Weakened interaction between exogenously expressed Myc-Lats2 with Flag-Siah2^RM^ in Zyxin-knockdown HEK293T cells. (**f**,**g**) MDA-MB-231 cells were treated with 5 μM MG132 and 5 ng ml^−1^ TGF-β for 12 h. Endogenous interaction between Lats2 and Siah2 was analysed by coimmunoprecipitation (**f**) and quantification of Siah2 (normalized to Lats2) protein levels (**g**). Ratio *t*-test was applied. All data are mean of *n*=3 independent experiments. All error bars indicate s.d. *P<0.05.

To validate our scenario, we first examined the interaction between Lats2 and Zyxin. Immunofluorescence showed that ectopic expressed Zyxin had relatively strong intensity of colocalization with Lats2 at cell membrane (Fig. 4a). Consistent with the immunofluorescence result, endogenous Zyxin was co-immunoprecipitated in Lats2 immunoprecipitated complex and vice versa (Fig. 4b). Ectopic expressed Zyxin-V5 was also found in ectopic expressed Myc-Lats2 immunoprecipitants (Fig. 4c). Further analysis of the binding region(s) showed that C-terminal (379-572) of Zyxin bound to Lats2 (Fig. 4d). Next, we examined the interaction between Zyxin and Siah2. Due to the highly unstable property of Siah2, we used Flag-Siah2^RM^ as bait, and found that Zyxin associated with Siah2 strongly (Fig.4e). To confirm the precise binding region(s) between Zyxin and Siah2, a series of deletion mutants were employed to the binding experiments. The results revealed that the C-terminal (379-572) of Zyxin bound to Siah2 (Fig. 4f) and the N-terminal (1-133) of Siah2 bound to Zyxin (Fig. 4g). Since Zyxin (379-572) interacts with both Siah2 and Lats2 (Fig. 4h), we may expect that Zyxin may form a complex with both Lats2 and Siah2. To further validate this hypothesis, we overexpressed Myc-Lats2, Flag-Siah2^RM^ and Zyxin-V5 in HEK293T cells, we first immunoprecipitated with anti-Flag antibody, then eluted the immunoprecipitated complex with Flag peptide, followed by a second immunoprecipitation from the elution with anti-Myc antibody (Fig. 5a). The result showed that Zyxin simultaneously interacted with both Lats2 and Siah2 (Fig. 5b), indicating that Zyxin-Lats2–Siah2 form a ternary complex *in vivo*. Then we asked whether Zyxin-Siah2–Lats2 ternary complex had a physiological function. We performed binding experiments and showed that Siah2-Lats2 binding intensity was dramatically increased along with increased Zyxin expression levels (Fig. 5c,d). Consistently, ectopic expressed Lats2 were found to be downregulated by Zyxin in a dosage-dependent manner, but this effect was abrogated by Siah2^RM^ (Supplementary Fig. 2a). However, knockdown of endogenous Zyxin potentiated dissociation of Siah2–Lats2 complex (Fig. 5e), indicating that Siah2 interacts with Lats2 not only in a Zyxin but also its dose-dependent manner. We further examined this effect at endogenous level by treating the cells with TGF-β. The result showed that the interaction between Lats2 and Siah2 was significantly enhanced in response to TGF-β treatment (Fig. 5f,g), suggesting that Zyxin may responsible for Lats2 degradation complex formation *in vivo*, which consistent with our previous data (Fig. 1e,f). Taken together, we conclude that Zyxin forms a functional ternary complex with Siah2 and Lats2, thus enhances their binding and promotes Siah2-induced Lats2 degradation.

Since Lats1 and Lats2 are homologous to *Drosophila* Warts^26^,^27^, we expected that Zyxin may similarly regulate Lats1. Indeed, Zyxin interacted with Lats1 (ref. 28) and induced its downregulation (Supplementary Fig. 2b), suggesting that Zyxin may involve in the regulation of both of Lats1 and Lats2 through identical mechanisms. Since Zyxin was previously reported having function of stabilizing actin filaments^29^,^30^,^31^, we wonder whether Zyxin-induced Lats2 destabilization is relevant to actin polymerization. The results showed that disruption of actin filaments by Cytochalasin D neither affected Siah2–Lats2 binding intensity (Supplementary Fig. 3a,b) nor Zyxin or TGF-β-induced Lats2 destabilization (Supplementary Fig. 3c–f), indicating that the effect of Zyxin on Lats2 is not relevant to the function of Zyxin in regulation of actin filaments. However, although Cytochalasin D could not block TGF-β-induced Lats2 destabilization, it could completely attenuate TGF-β-induced Yap dephosphorylation and nuclear translocation (Supplementary Fig. 3e–g), indicating that Yap activity could be influenced by mechanical perturbations in an actin-dependent fashion independent of Lats activity, which is consistent with previous report^16^,^32^,^33^,^34^,^35^.

### Zyxin is required for cell migration and tumour growth

**Figure 6:**
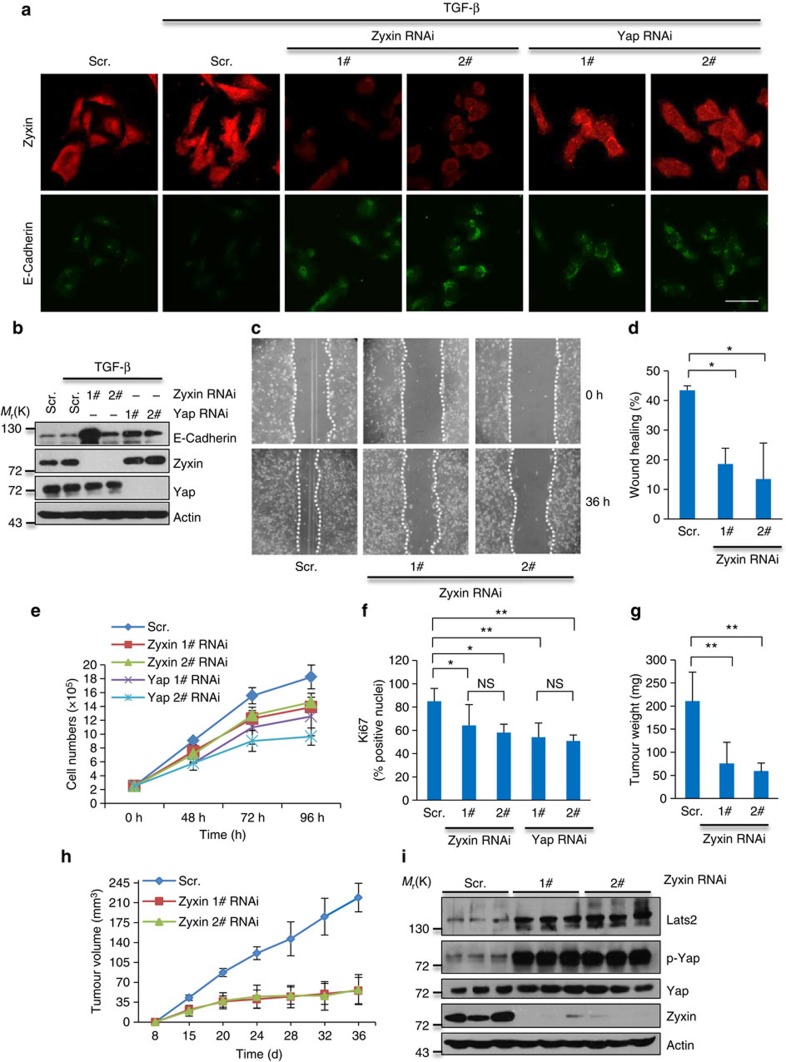
Zyxin is required for cell migration and tumour growth. (**a**,**b**) Zyxin-knockdown and Yap-knockdown MDA-MB-231 cells were treated with 5 ng ml^−1^ TGF-β for 12 h and stained with antibody against Zyxin and E-Cadherin (**a**), cell lysates were subjected for immunoblotting with indicated antibodies (**b**). Scale bars, 50 μm. (**c**,**d**) Migration of Scramble and Zyxin-knockdown MDA-MB-231 cells into the wound was monitored (**c**) and quantitative analysis of wound healing (**d**). The area was quantified by ImageJ software. (**e**) Cell proliferation rate of MDA-MB-231 cells bearing short hairpin RNAs (shRNAs) against indicated genes. (**f**) Quantitative analysis of Ki67-positive rate of MDA-MB-231 cells bearing shRNAs against indicated genes. (**g**,**h**) Tumour weight (**g**) and tumour growth curves (**h**) of mice with subcutaneous injection of MDA-MB-231 cells with or without knockdown of Zyxin. (**i**) Western blotting analysis of xenograft tumour tissues with the indicated antibodies. Data in **e** and **f** are the mean of *n*=3 independent experiments. *n*=5 mice per group in **g** and **h**. All error bars indicate s.d. *P<0.05, **P<0.01, NS, not significant. Two-tailed, unpaired Student's *t*-test.

EMT plays an important role in the regulation of embryonic development, as well as in various pathological conditions including fibrosis and cancer^36^,^37^,^38^,^39^. TGF-β has been clearly shown as a major inducer of this process^39^. TGF-β treated MDA-MB-231 cells showed reduction of E-cadherin expression whereas knockdown of Zyxin, as well as Yap, abrogated such effect (Fig. 6a,b), indicating that TGF-β-induced EMT was blocked by Zyxin knockdown. Indeed, Zyxin-knockdown MDA-MB-231 cells showed impaired cell motility (Fig. 6c,d), indicating that Zyxin is required for MDA-MB-231 cells' migration. This result is consistent with previous studies that Zyxin controls migration in MDCK cells^40^ and another LIM domain containing protein, LPP, a Zyxin homologue, controls TGF-β induced migration in breast cancer cells^41^. Intriguingly, all phenotypes including the Hippo target gene expression and EMT of Zyxin-knockdown cells were similar with the phenotype of Yap-knockdown cells (Fig. 1g and Fig. 6a,b), supporting the notion that Zyxin may play a role in these processes associating with Yap.

To investigate the biological relevance of Zyxin, we compared and assessed the properties of Zyxin-knockdown with Yap-knockdown in regulation of cell proliferation. Both Zyxin-knockdown cells and Yap-knockdown cells showed decreased cell proliferation rate (Fig. 6e) and reduced Ki67-positive cells (Fig. 6f). By using a xenograft mouse model, we show that Zyxin plays an important role in promoting tumourigenesis, as Zyxin knockdown led to a decrease in tumourigenicity of MDA-MB-231 cells (Fig. 6g,h). Mice bearing Zyxin-silenced cells showed average 3.9-fold and 3.2-fold decrease in tumour weight (Fig. 6g) and volume (Fig. 6h) compared with scramble group, respectively. Immunoblotting analysis of the implanted tumour tissues revealed increased Lats2 and p-Yap levels in Zyxin-knockdown tumours (Fig. 6i). This therefore suggests that Zyxin may play an important role in tumourgenesis through the Hippo signalling pathway. This is consistent with previous studies of Zyx102 in *Drosophila* as its loss reduces Yorkie activity and organ growth^29^,^42^.

### Zyxin expression correlates with tumour malignancy

**Figure 7:**
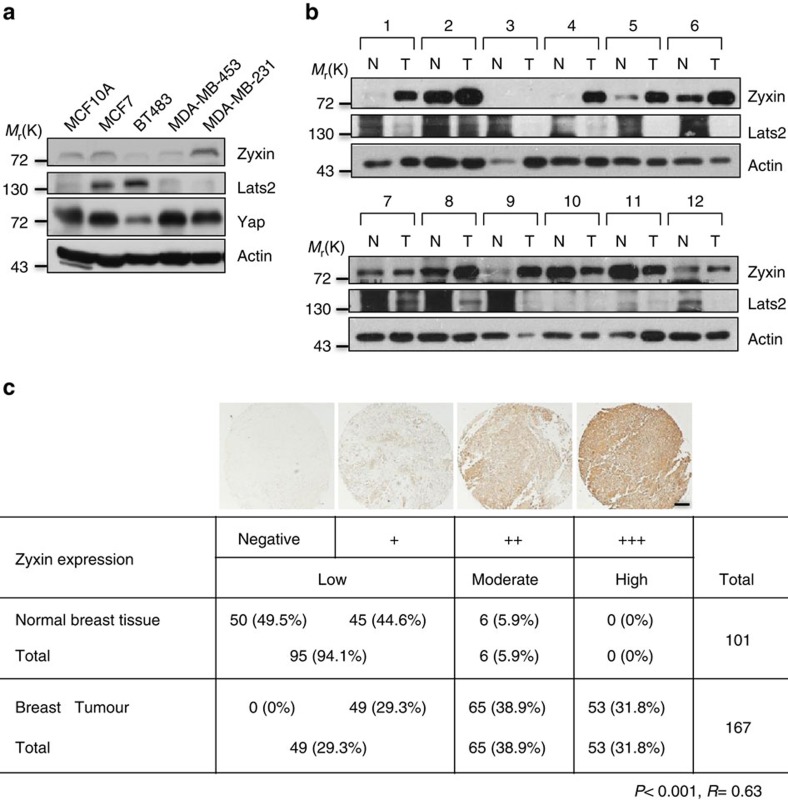
Zyxin is upregulated in human breast cancer. (**a**) Expression of Zyxin, Lats2 and Yap in human breast cell lines. (**b**) Twelve pairs of fresh frozen breast cancer tissues and corresponding tissues were lysed, and cell lysates were immunoblotted with the indicated antibodies. (N, normal tissues; T, tumour tissues). (**c**) Zyxin protein is upregulated in human breast cancer. Immunohistochemical staining of Zyxin in representative normal breast tissues and breast cancer tissues on the tissue microarrays. Scale bars, 250 μm. (Brown colour indicates positive immune reaction.) Statistical significance was determined by *χ*^2^ test. *R* is the correlation coefficient.

Our results shown in Fig. 6g–i demonstrate Zyxin's ability to promote xenograft tumour growth and lend support to the hypothesis that Zyxin may function as an oncogene *in vivo*. We first examined the expression of Zyxin in a panel of breast cell lines. Those cell lines such as MDA-MB-231 has higher malignancy, which has a high-expression level of Zyxin and low-expression level of Lats2. This negative correlation between Zyxin and Lats2 also showed in other breast cell lines (Fig. 7a) and in majority of the breast tissues that we examined (Fig. 7b). Besides, Yap expression level also negatively correlated with Lats2 (Fig. 7a), this is possibly due to the phosphorylation on Yap by Lats2 could promote its degradation^43^. To further study the role of Zyxin in human cancer, we examined the expression level of Zyxin in human breast cancer tissue microarrays. Notably, moderate and high expression of Zyxin was observed in 70.7% (118 out of 167) of breast tumours compared with 5.9% (6 out of 101) of normal breast tissues, indicating a dramatic significant positive correlation between breast tumourigenesis and Zyxin expression (Fig. 7c). Baseline characteristics were compared among Zyxin low-expression, moderate-expression and high-expression patients. Analysis of the relationship between Zyxin immunostaining and clinicopathological parameters showed that higher immunostaining intensity was significantly correlated with histological stage and lymph node metastasis, but not significantly correlated with age or tumour size (Table 1). The results demonstrate that Zyxin may be a potential prognostic marker in breast cancer patients.

## Discussion

Intrinsic and extrinsic factors coordinately regulate proper cell fate and tissue size. How upstream signals that regulate the Hippo pathway have been widely studied, cross-talks between the Hippo pathway and other signalling pathways have emerged^15^,^44^,^45^,^46^. TGF-β signalling pathways has been reported to be dependent on the Hippo pathway activity and the Hippo pathway kinase Lats regulates SMAD localization via Taz/Yap (refs 19, 20), but how Taz/Yap response to TGF-β stimuli remains elusive. Previous studies have already shown that TGF-β may contribute to advanced malignancies through its pro-oncogenic effects, such as pro-proliferation, pro-metastasis, promotion of angiogenesis and anti-immune response^47^,^48^,^49^,^50^. Similarly, deactivation of the Hippo pathway contributes to tumourigenesis and metastasis^5^,^6^,^12^ and downregulation of the components of the Hippo pathway has been indeed observed in various types of cancer^13^,^14^,^15^,^16^. These phenomena indicate that the cross-talk between the Hippo pathway and TGF-β signalling pathway may together play a pro-oncogenic role in malignant tumours.

**Figure 8:**
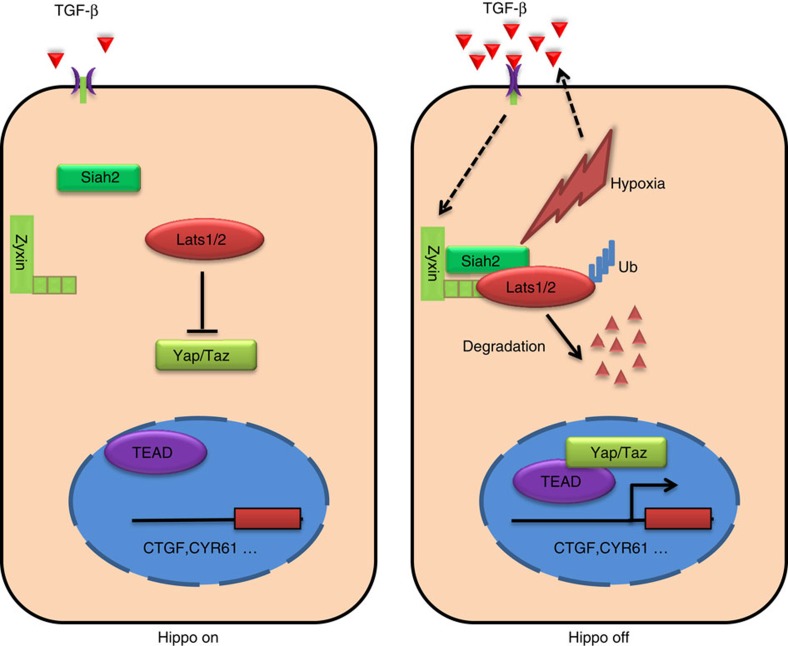
A proposed model of TGF-β in regulation of the Hippo pathway. Hypoxia activates Siah2 and enhances the secretion of TGF-β. Zyxin functions as a scaffold protein that is upregulated in response to TGF-β stimuli, thus forming a ternary complex with Siah2 and Lats2, thereby facilitating Siah2-Lats2 binding and promoting Lats2 degradation and coordinately activation of Yap.

Hypoxia is an important microenvironment factor that contributes to tumour progression and that is also a common feature in solid tumours^21^. Hypoxic cells can benefit from Yap activation mainly due to hypoxia-induced activation of Siah2 and subsequent degradation of Lats2 (ref. 15), whereas tumour cells within the hypoxic microenvironment may also benefit from increased secretion of TGF-β in an autocrine (Fig. 1a,b) and paracrine way^51^. It is possible that, the development of a hypoxic microenvironment during tumour growth leads to both of Siah2 and Zyxin activation through hypoxia and hypoxia-induced upregulation of TGF-β, respectively, which results in increased binding intensity of Lats2–Siah2 complex, thus allowing the efficient degradation of Lats2 and the activation of Yap (Fig. 8). The xenograft experiments further validate our notion that Zyxin ablation could indeed deactivate Hippo signalling in planting tumours (Fig. 6h). Importantly, overexpression of Zyxin was observed within human breast cancer tissues and its expression level was associated with tumour progression and metastasis (Fig. 7c and Table 1). Consistent with our finding, recent research also found that Zyxin may have a pro-oncogenic role in regulation of tissue growth^29^,^42^. Thus, Zyxin activation may play a critical role in regulating Yap activation during tumourigenesis. Further investigations of how Hypoxia, TGF-β and the Hippo pathway are mutually regulated may shed light on therapeutic strategies against cancer.

## Materials and methods

### Cell culture, reagents and expression constructs

Human epithelial HeLa, HEK293T and MDA-MB-231 cell lines were purchased from the American Type Culture Collection (ATCC) and were cultured in DMEM (GIBCO) supplemented with 10% foetal bovine serum (FBS, Hyclone) and 1% penicillin/streptomycin at 37 °C under 5% CO_2_. The cell lines have been tested free for Mycoplasma contamination. Hypoxic conditions were created by culturing cells in the hypoxia chamber (Billups-Rothenberg) flushed with 1% O_2_, 5% CO_2_ and 94% N_2_ mixture gas. Human recombinant TGF-β1 was purchased from Peprotech. Plasmids were transfected with Lipo2000 according to the manufacturer's instruction. SB431542 (S1067) was purchased from Selleck Chemicals. Cytochalasin D was purchased from Sigma. Expression plasmids of Siah2 and Zyxin were generated by PCR and cloned in pFlag-CMV-4, pGEX-4T-1 or pEF1/V5-His expression vectors. Myc-Lats2 plasmid was constructed by insertion of Lats2 cDNA in frame into the pcDNA3.0 vector. All Siah2 and Zyxin mutant constructs were created using the Easy Mutagenesis System (TransGen Biotech). All the plasmids were confirmed by DNA sequencing.

### Immunoblotting

Cells were lysed in lysis buffer (150 mM NaCl 20 mM Tris, pH 7.4, 1 mM EDTA, 1 mM EGTA, 1 mM Na_3_VO_4_, 2.5 mM Sodium pyrophosphate, 10% glycerol, 1% NP-40 and protease inhibitors). Equivalent cell lysates were subjected to SDS–polyacrylamide gel electrophoresis, and transferred to nitrocellulose membranes, then were blocked with 5% non-fat milk or 5% BSA (Dingguo changsheng Biotechnology) for 1 h at room temperature. The membranes were then probed with the indicated primary antibodies, followed by the appropriate HRP-conjugated secondary antibodies (KPL). Signals were visualized with chemiluminescence kits (Engreen Biosystem). The following antibodies were used: antibodies against Lats2 (1:1000, Abcam, ab70565), Siah2 (1:500, Novus, NB110-88113, clone 24E6H3), Zyxin (1:10000, Epitomics, 3586-1, EPR4302), Actin (1:10000, Sigma, A5441, clone AC-15), CTGF (1:500, Santa Cruz, sc-14939), CYR61 (1:500, Santa Cruz, sc-13100), p-S127-Yap (1:1000, Cell Signalling, 4911), Yap (1:1000, Epitomics, 2060-1, clone EP1674Y), Flag (1:1000, Sigma, F1804, clone M2), Myc (1:10000, Proteintech, 60003-2-Ig), V5 (1:1000, sungenebiotech, KM8006, clone 4D6), HA (1:1000, Abmart, M20003, clone 26D11), HIF1α (1:1000, Epitomics, 2015-1, clone EP1215Y) and Ubiquitin (1:1000, Enzo Life Sciences, PW8805, clone FK1). The ImageJ software ( http://rsbweb.nih.gov/ij/download.html) was used for image analyses and the quantification results were normalized to the loading control. The uncropped images of all immunoblots were shown in Supplementary Fig 5.

### Immunoprecipitation

Cells were harvested and lysed in 0.5 ml lysis buffer plus protease inhibitors (Roche) for 1 h on rotor at 4 °C. After 12,000*g* centrifugation for 15 min, the lysates were incubated with 2 μg specific antibody overnight at 4 °C, 30 μl protein A/G-agarose beads (Santa Cruz, SC-2003) were washed with lysis buffer and then added for additional 3 h. Thereafter, the beads were washed five times with lysis buffer and boiled with loading buffer for 5 min and subjected to SDS–polyacrylamide gel electrophoresis for analysis. Flag-peptides were used for elution were purchased from ChinaPeptides. The following antibodies were used for immunoprecipitation: antibodies against Zyxin (Epitomics, 3586-1, EPR4302), Flag (Sigma, F1804, clone M2), Myc (Santa Cruz, sc-40, clone 9E10) and Lats2 (Abcam, ab70565).

### Glutathione S-transferase (GST) pull-down and Ni-resin pull-down assays

Recombinant GST-Siah2 proteins were produced in *Escherichia coli* BL21 (DE3) cells and purified with Glutathione Sepharose 4B (GE healthcare). Zyxin-V5-His and its mutants were ectopically expressed in HEK293T cells and purified by Ni-NTA resin (GE Healthcare) according to standard protocols. GST (10 μg) or GST fusion proteins were incubated with Glutathione Sepharose 4B for 2 h at 4 °C, followed by incubation with cell extracts at 4 °C overnight. After 2,800*g* centrifugation, supernatants were collected as input and the argrose beads were then extensively washed five times each with 1 ml lysis buffer and boiled with SDS loading buffer for 5 min then analysed by western blotting.

### *In vivo* ubiquitination assay

HEK293T cells were transfected with plasmids expressing, Myc-Lats2, HA-ubiquitin, Zyxin-V5 alone or together with Flag-Siah2^RM^. Twenty-four hours after transfection, cells were firstly treated with 10 μM MG132 (Selleckchem S2619) for 6 h then harvested. Using cold PBS to wash the cells then lysed in 200 μl of denaturing buffer (150 mM Tris-HCl pH 7.4, 1% SDS) by sonication and boiling for 10 min. Additional lysis buffer was added to the Lysates to 1 ml and incubated with 2 μg anti-c-Myc antibody followed by immunoprecipitation with protein A/G-agarose beads. The precipitants were subjected to western blotting with anti-HA or anti-Myc antibody. For endogenous Lats2 ubiquitination assay, Scramble or Zyxin-knockdown cells were treated with 10 μM MG132 for 6 h before harvest. Lysates were incubated using 2 μg anti-Lats2 antibody and analysed by western blotting using anti-Lats2 or anti-Ubiquitin antibodies.

### Immunofluorescence microscopy

Cells were grown to 40–60% confluence on coverslips. The cells were fixed with 4% paraformaldehyde (Dingguo changsheng Biotechnology) for 10 min at room temperature followed by washing three times with PBS. The cells were permeabilized with 0.1% Triton X-100 with 4,6-diamidino-2-phenylindole for 5 min at 4 °C. The coverslips were blocked by goat serum for 30 min at room temperature and then incubated with FITC- or CY3-conjugated secondary antibodies (Invitrogen, 1:1,000) for 1 h at room temperature. The coverslips were then washed three times with PBS and mounted. The following antibodies were used for immunofluorescence: antibodies against Zyxin (1:200, Epitomics, 3586-1, EPR4302), Yap (1:100, Epitomics, 2060-1, clone EP1674Y) and E-cadherin (1:100, BD Biosciences, 610182, clone 36/E-Cadherin). FITC-conjugated Phalloidin was purchased from ThermoFisher. Cell images were captured with confocal microscope (Leica). Colocalization correlation coefficient was calculated by using ImageJ software.

### Lentiviral production and infection

Lentiviral packaging plasmids psPAX2 and pMD2.G were co-transfected with the PLKO.1 backbone plasmid into HEK293T cells for virus production. Cells were selected in 2.5 μg ml^−1^ puromycin (Sangon Biotech PJ593) in culture medium. The oligonucleotide pairs used as follows: Yap 1# and Yap 2# were described previously^15^. Zyxin 1#: (5′- CCGGGAAGGTGAGCAGTATTGATTTCTCGAGAAATCAATACTGCTCACCTTCTTTTTG -3′ and 5′- AATTCAAAAAGAAGGTGAGCAGTATTGATTTCTCGAGAAATCAATACTGCTCACCTTC -3′); Zyxin 2#: (5′- CCGGCTTCCACATGAAGTGTTACAACTCGAGTTGTAACACTTCATGTGGAAGTTTTTG -3′ and 5′- AATTCAAAAACTTCCACATGAAGTGTTACAACTCGAGTTGTAACACTTCATGTGGAAG -3′).

### RNA isolation and real-time PCR

Trizol reagent (Roche) was used for extraction of total RNA from cultured cells. cDNA was reverse transcripted using oligo (dT) and subjected to real-time PCR in the presence of SYBR Green PCR-Mix (Roche). mRNA relative abundance was calculated by normalization to *ACTB* mRNA. The following primers were used for RT-qPCR: *CYR61* (5′- GGTCAAAGTTACCGGGCAGT -3′ and 5′- GGAGGCATCGAATCCCAGC -3′); *CTGF* (5′- ACCGACTGGAAGACACGTTTG -3′ and 5′- CCAGGTCAGCTTCGCAAGG -3′); *Lats2* (5′- ACCCCAAAGTTCGGACCTTAT -3′ and 5′- CATTTGCCGGTTCACTTCTGC -3′); *TGF-β (*5′- CCAACTATTGCTTCAGCTCCA -3′ and 5′- TTATGCTGGTTGTACAGGG -3′); *ACTB* (5′- CATGTACGTTGCTATCCAGGC -3′ and 5′- CTCCTTAATGTCACGCACGAT -3′). Data were analysed from three independent experiments and shown as the average mean±s.d.

### Quantitative analysis of activated human TGF-β1

Cells were seeded on 60 mm dish in 3 ml DMEM supplemented with 10% FBS (HyClone). 24 h later, cells were washed by FBS-free DMEM for four times and incubated under normoxic or hypoxic conditions for another 12 h. Culture medium was collected and the activated human TGF-β1 was determined by ELISA kit (Multisciences Biotech).

### Wound healing assay

Cells were plated in 6-well dishes in triplicates. Once cells were grown to confluence, the cell monolayer was scratched by a P100 pipet tip, then were washed twice with serum-free DMEM and incubated with 2 ml of serum-free DMEM for another 36 h. The percentage of wound closure was calculated by using ImageJ software.

### Cell proliferation assay

Zyxin-knockdown MDA-MB-231 cells (2 × 10^5^) were seeded on 6-well plate in triplicates. Cell growth was measured through counting cell numbers at indicated time points.

### *In vivo* tumourigenesis study

All animal experiments were approved by the Institutional Animal Care and Use Committee at College of Life Sciences at Nankai University. MDA-MB-231 breast cancer cells (2 × 10^6^ in 100 μl PBS) were injected subcutaneously into armpit of 6- to 8-week-old female nude mice (BALB/c-nu). The tumour size was measured every 4 days after a week post of the implantation and the tumour volume was calculated by using the formula *V*=0.5 × *L* × *W*^2^ (*V*, volume; *L*, length; *W*, width). The xenograft tumours were surgically removed after 36 days, then weighted and photographed. No statistical method was used to predetermine sample size for each group. The experiments were not randomized.

### Tissue microarray and immunohistochemistry

One hundred and sixty-seven analysable cases of breast carcinoma and 101 analysable cases of normal breast tissue on the tissue microarray slide (US Biomax) were analysed. The slides were treated as described previously^15^. The anti-Zyxin (1:150, Epitomics, 3586-1, EPR4302) antibody was used for immunohistochemistry. Signal was visualized with a DAB Substrate Kit (MaiXin Bio). Protein expression levels of all the samples were scored as four grades (negative, +, ++, +++) according to the percentage of immunopositive cells and immunostaining intensity. Grades represent: negative and+were defined as low expression, ++ was defined as moderate expression, +++ was defined as high expression. Test of anti-Zyxin antibody for immunohistochemistry was shown in Supplementary Fig. 4.

### Statistics and repeatability of experiments

Statistical comparisons were made using the two-tailed paired ratio *t*-test using Prism or unpaired Student's two-tailed *t*-test for two data sets using Excel (Microsoft). All error bars indicate s.d. For statistical tests, *P*<0.05 was set as the criterion for statistical significance. The correlation coefficient *R* was calculated by comparison between Zyxin expression level ≤+ and level⩾++ in normal and tumour tissues. The *χ*^2^ test was used for analysis of the significance of Zyxin expression in normal and tumour tissues and the clinicopathological parameters. These experiments were repeated at least three times. The investigators were not blinded to allocation during experiments and outcome assessment.

## Additional information

**How to cite this article:** Ma, B. *et al*. Zyxin-Siah2–Lats2 axis mediates cooperation between Hippo and TGF-β signalling pathways. *Nat. Commun.* 7:11123 doi: 10.1038/ncomms11123 (2016).

## Supplementary Material

Supplementary Information Supplementary Figures 1-5

## Figures and Tables

**Table 1:** Patient characteristics based on Zyxin expression.

**Variables**	***n*****=167**	**Zyxin expression**			***P*****-value**
		**Negative/low**	**Moderate**	**High**	
Age					0.859
>50 years	68	22	24	22	
≤ 50 years	99	27	41	31	
					
Tumour size					0.189
T1	12	3	5	4	
T2	116	38	43	35	
T3	19	3	12	4	
T4	20	5	5	10	
					
Stage					0.036
I	9	3	4	2	
lla	65	26	22	17	
llb	58	11	30	17	
lll	35	9	9	17	
					
Lymph node metastasis					0.025
N0	94	34	35	25	
N1/2	73	13	31	29	
