# Supplementary material for: Zyxin-Siah2–Lats2 axis mediates cooperation between Hippo and TGF-β signalling pathways
Source: Nat Commun. 2016 Mar 31;7:11123. doi: 10.1038/ncomms11123 (PMC4821889; doi:10.1038/ncomms11123)
Supplement: Supplementary Information — Supplementary Figures 1-5 [file ncomms11123-s1.pdf]

Supplementary Figure 1: Loss of Zyxin stabilizes Lats2 and activates Hippo signalling.

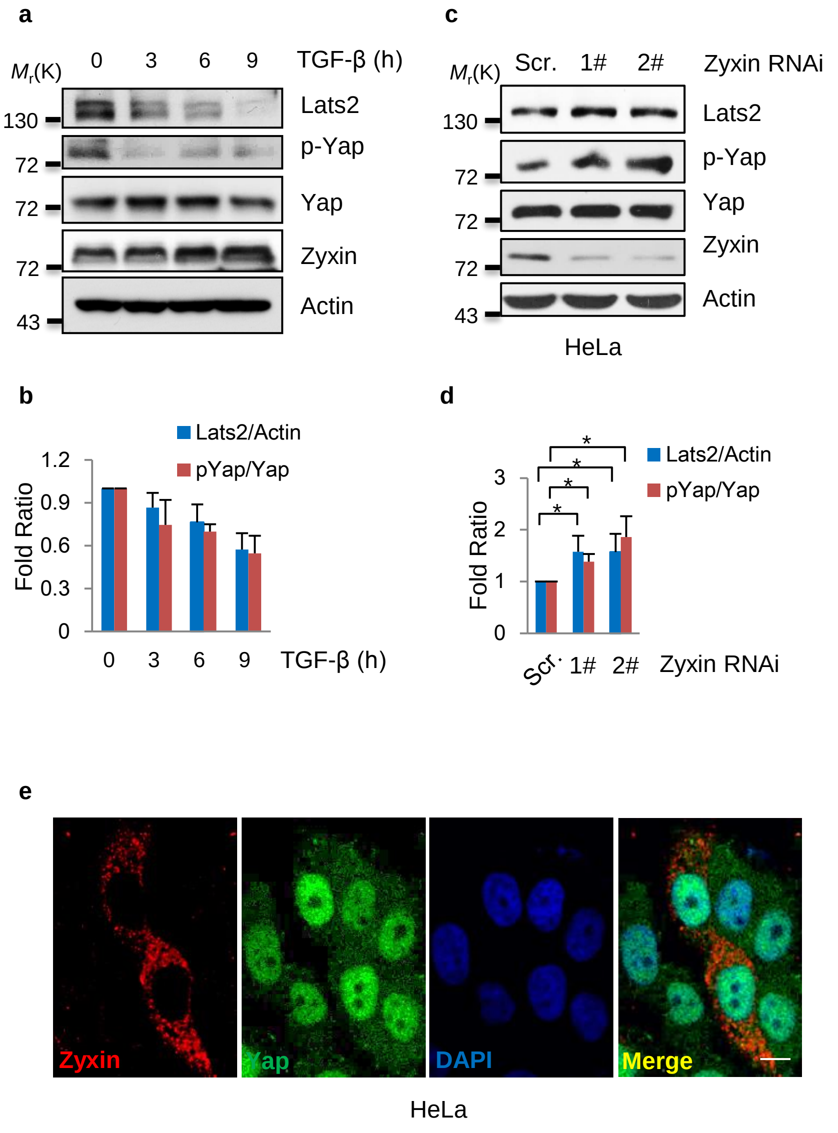

Supplementary Figure 1: Loss of Zyxin stabilizes Lats2 and activates Hippo Signalling.
(a, b) MDA-MB-231 cells were treated with 5 ng ml-1 TGF-β, collected at indicated the time points and then immunoblotted with the indicated antibodies **(a)** and quantification of Lats2 (normalized to actin) and p-Yap (normalized to Yap) protein levels **(b)**. Ratio t-test was applied. **(c, d)** HeLa cells stably expressed shRNA against Zyxin were collected for immunoblotting with indicated antibodies **(c)** and quantification of Lats2 (normalized to actin) and p-Yap (normalized to Yap) protein levels **(d)**. Ratio t-test was applied. **(e)** Zyxin-V5 overexpressed HeLa cells were stained with DAPI and antibodies against Yap and V5. Scale bars: 10 µm. All error bars indicate s.d.. ∗, $P < 0.05$.

Supplementary Figure 2: Zyxin potentiates Lats2 degradation.

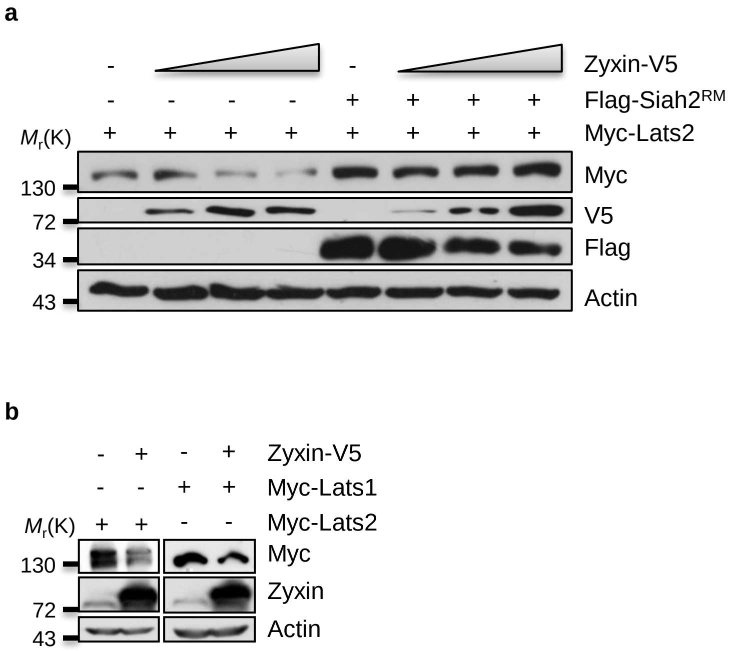

Supplementary Figure 2: Zyxin potentiates Lats2 degradation in a dosage dependent manner. **(a)** Lats2 degradation was dosage-dependent on Zyxin and this effect was blocked by the dominant negative mutant of Siah2. **(b)** Lats1/2 stability was regulated by Zyxin.

Supplementary Figure 3: Zyxin facilitates Siah2-Lats2 binding is independent of F-actin cytoskeleton.

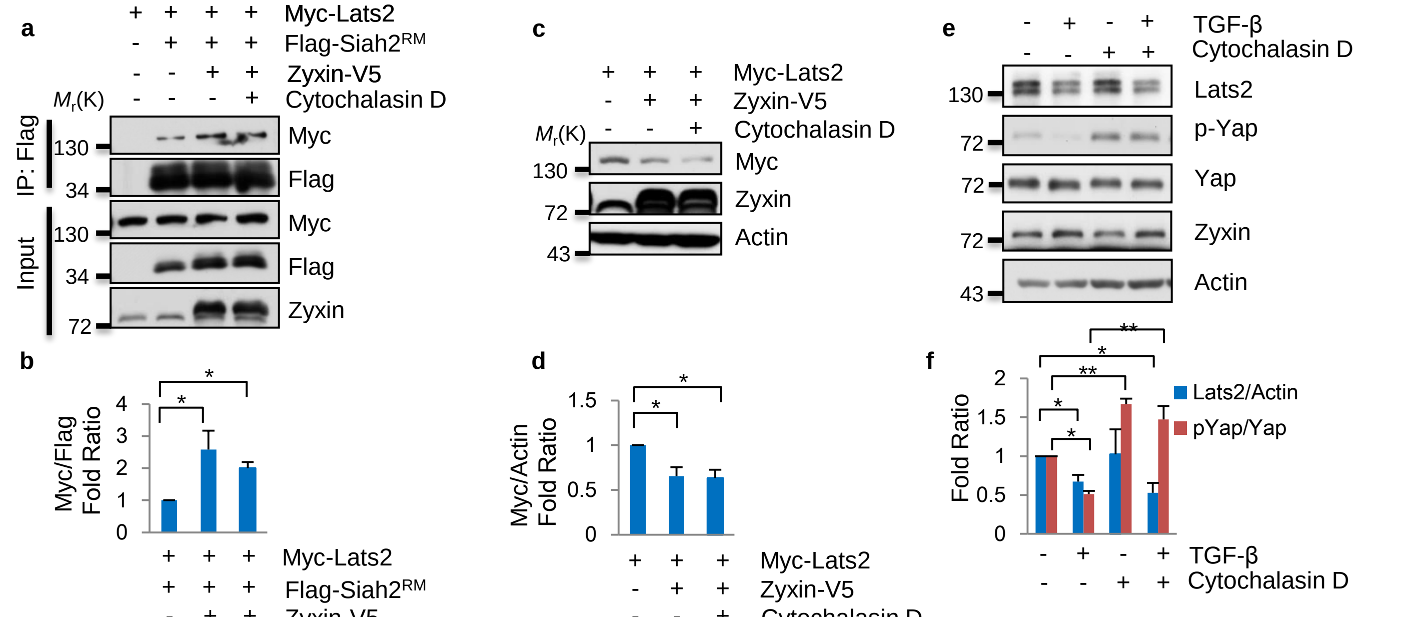

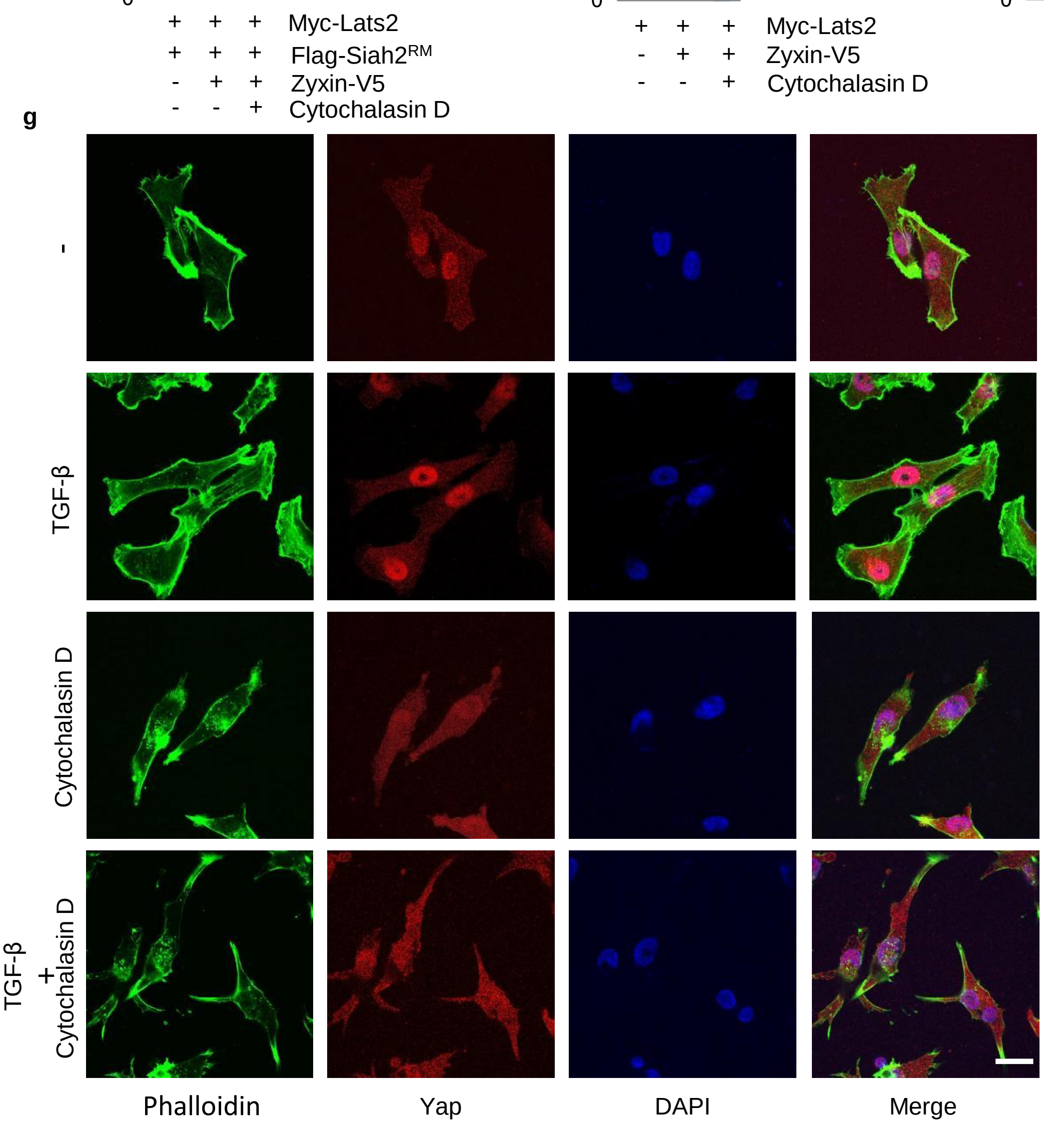

Supplementary Figure 3: Zyxin facilitates Siah2-Lats2 binding is independent of F-actin cytoskeleton.
(a, b) HEK293T cells were transfected with the indicated plasmids for 20 hours, then cells were treated with Cytochalasin D (0.25 μM) for another 5 hours. Cells were collected and subjected to coimmunoprecipitation **(a)** and quantification of Myc-Lats2 (normalized to Flag-Siah2RM) protein levels **(b)**. Ratio t-test was applied. **(c ,d)** Cells were transfected with the indicated plasmids for 20 hours, then cells were treated with Cytochalasin D (0.25 μM) for another 5 hours. Cells were collected and subjected to immunoblotting with the indicated antibodies **(c)** and quantification of Myc-Lats2 (normalized to Actin) protein levels **(d)**. Ratio t-test was applied. (e, f) MDA-MB-231 cells were treated with 5 ng ml-1 TGF-β for 10 hours ,then in the presence or absence of Cytochalasin D (0.25 μM) for another 5 hours. Cells were collected and subjected to immunoblotting with the indicated antibodies **(e)** and quantification of Lats2 (normalized to actin) and p-Yap (normalized to Yap) protein levels **(f)**. Ratio t-test was applied. **(g)** MDA-MB-231 cells in **(e)** were stained with DAPI, Phalloidin and antibody against Yap. Scale bars: 25 μm. All error bars indicate s.d.. *, $P < 0.05$; **, $P < 0.01$.

Supplementary Figure 4: Specificity evaluation of anti-Zyxin antibody by IHC analysis.

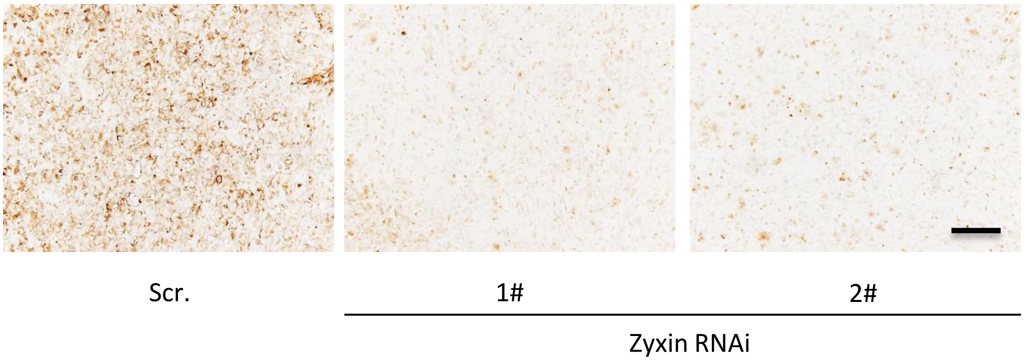

Supplementary Figure 4: Specificity evaluation of anti-Zyxin antibody by IHC analysis.
Zyxin knockdown xenograft tumor tissues were used to verify the specificity of Zyxin antibody for IHC. Scale bars: 50 μm.

Supplementary Figure 5: Uncropped images of key blots/gels.

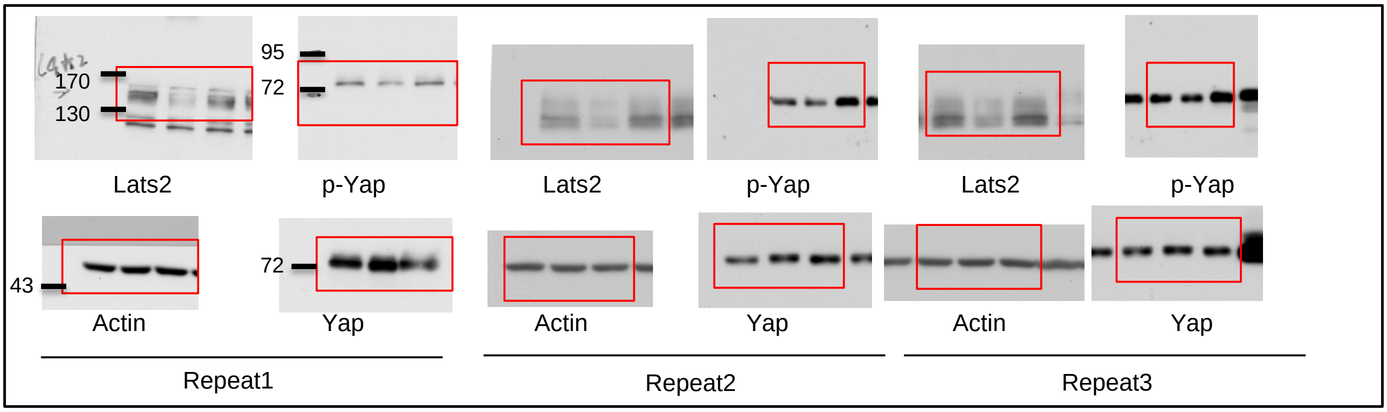

Figure 1c

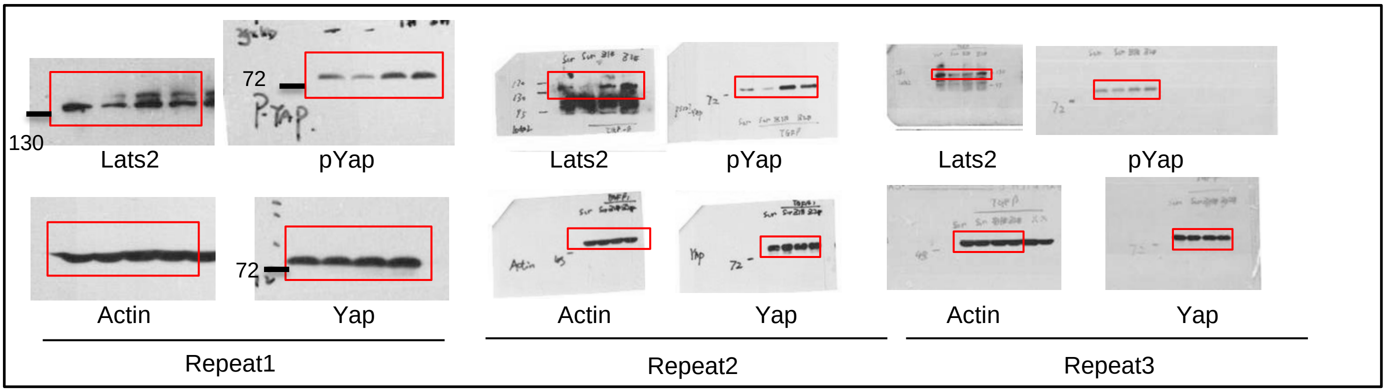

Figure 1e

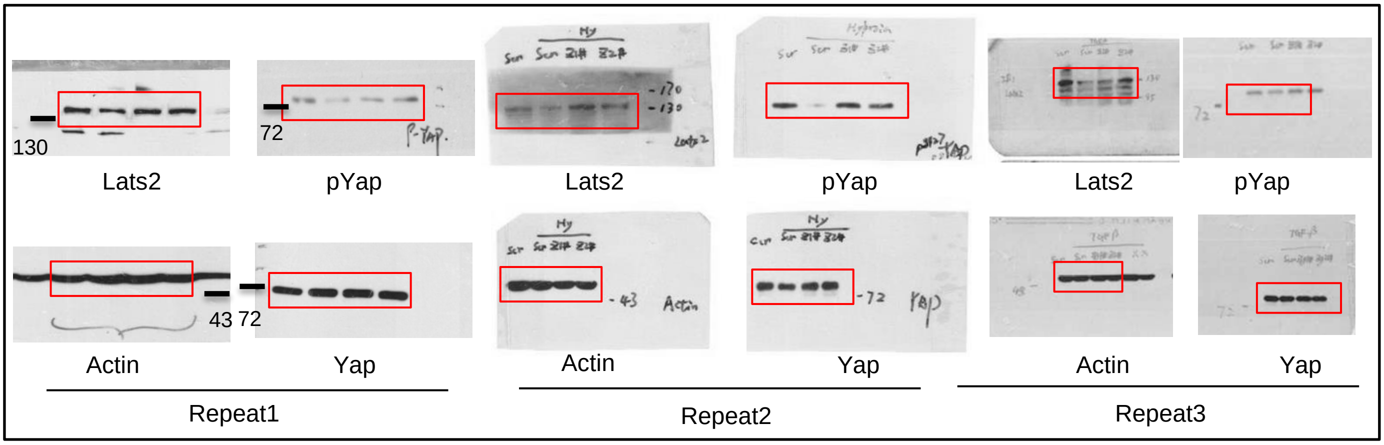

Figure 1h

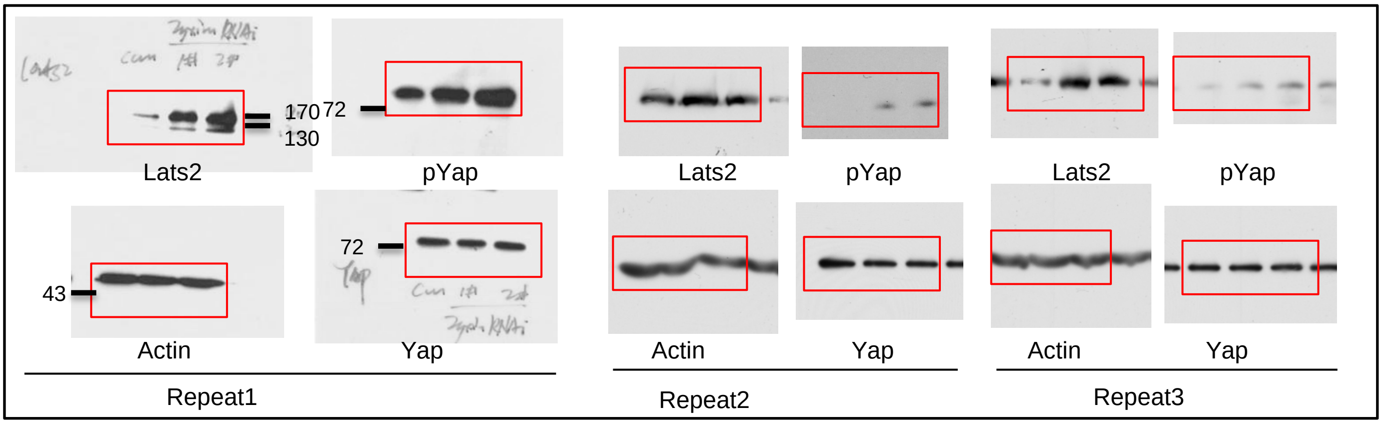

Figure 2a

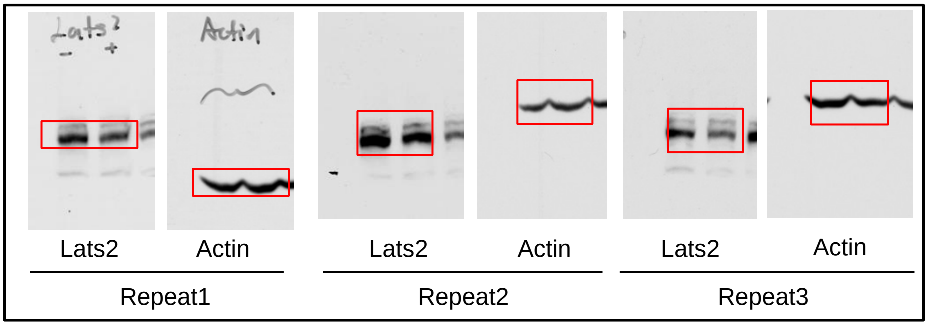

Figure 2c

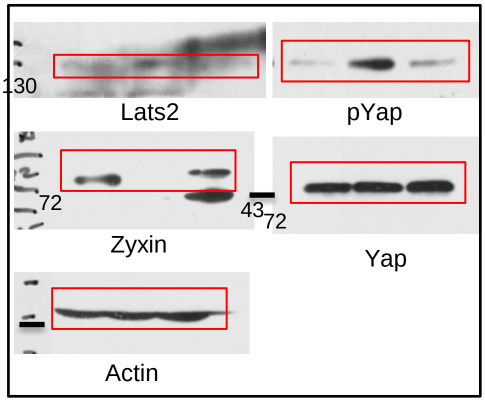

Figure 2e

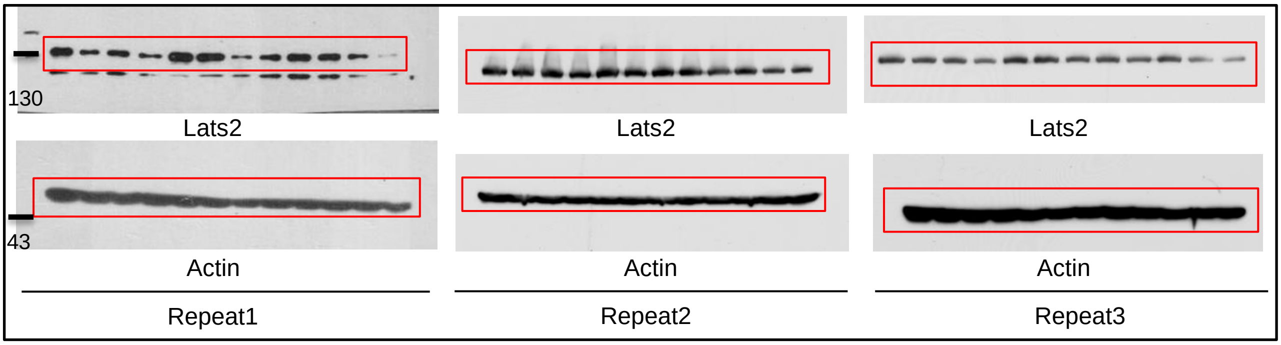

Figure 2f

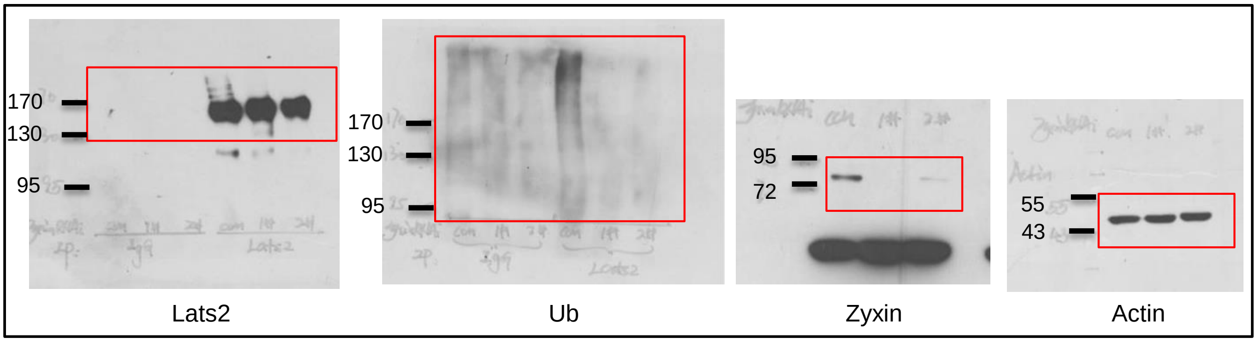

Figure 3a

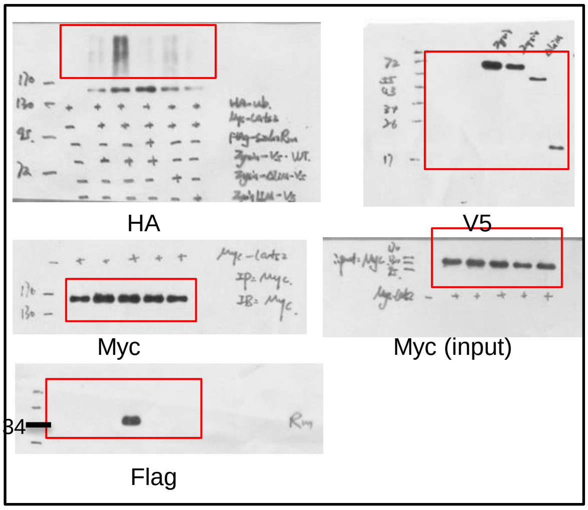

Figure 3b

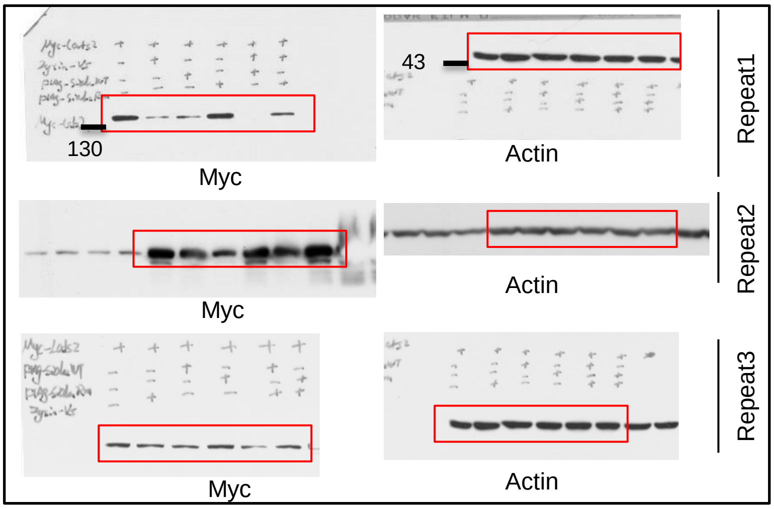

Figure 3c

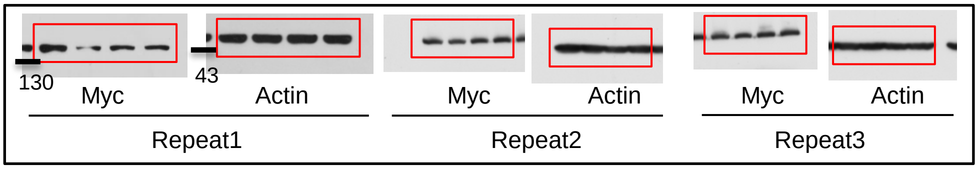

Figure 3e

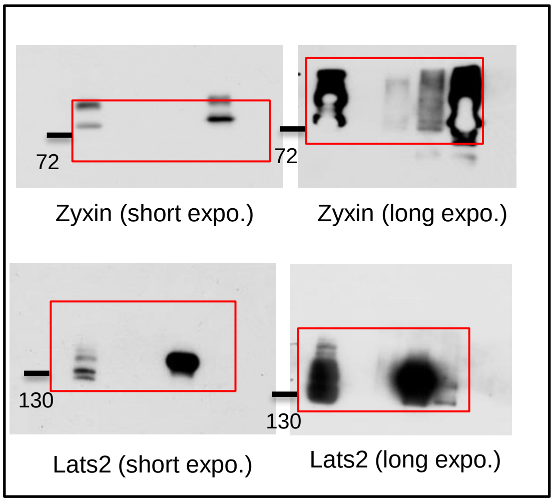

Figure 4b

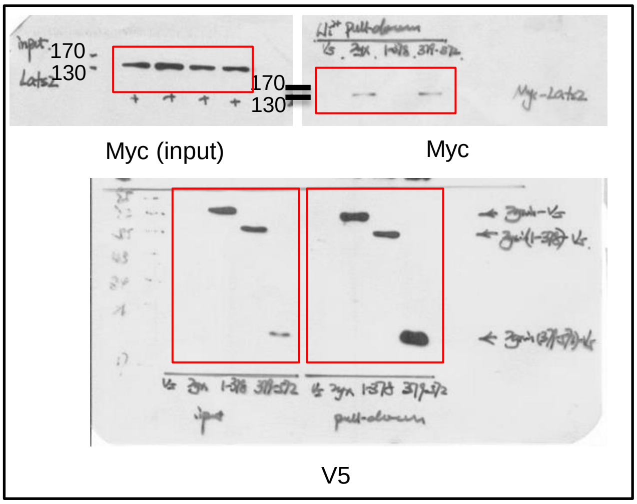

Figure 4d

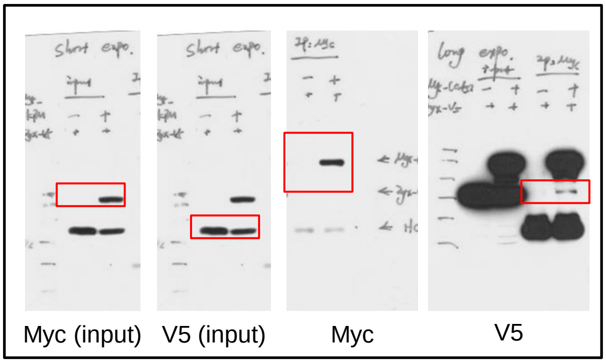

Figure 4c

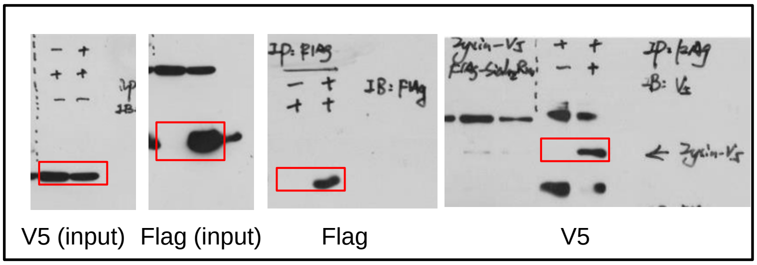

Figure 4e

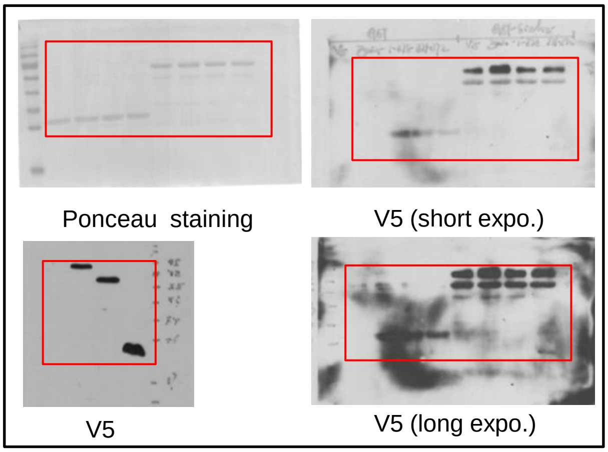

Figure 4f

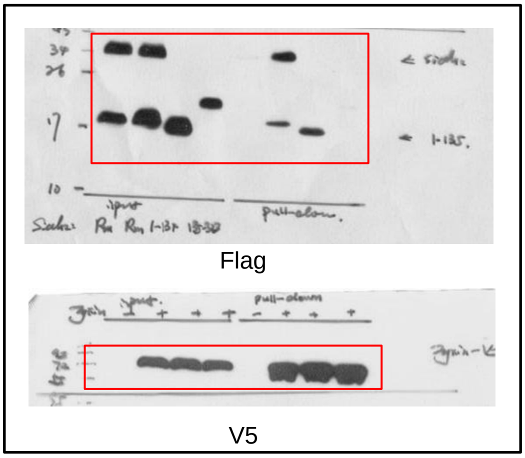

Figure 4g

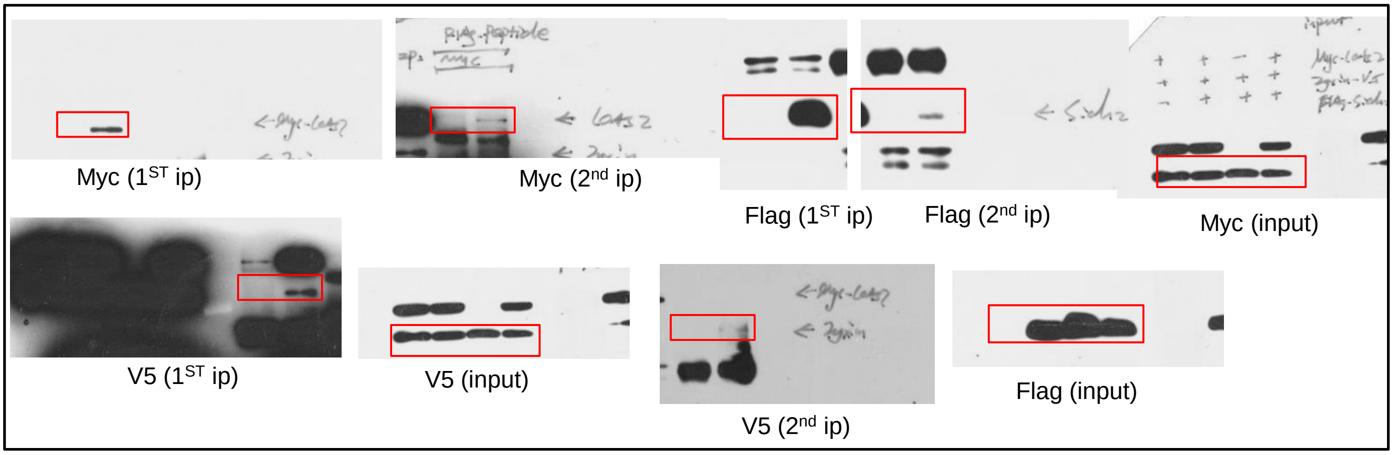

Figure 5b

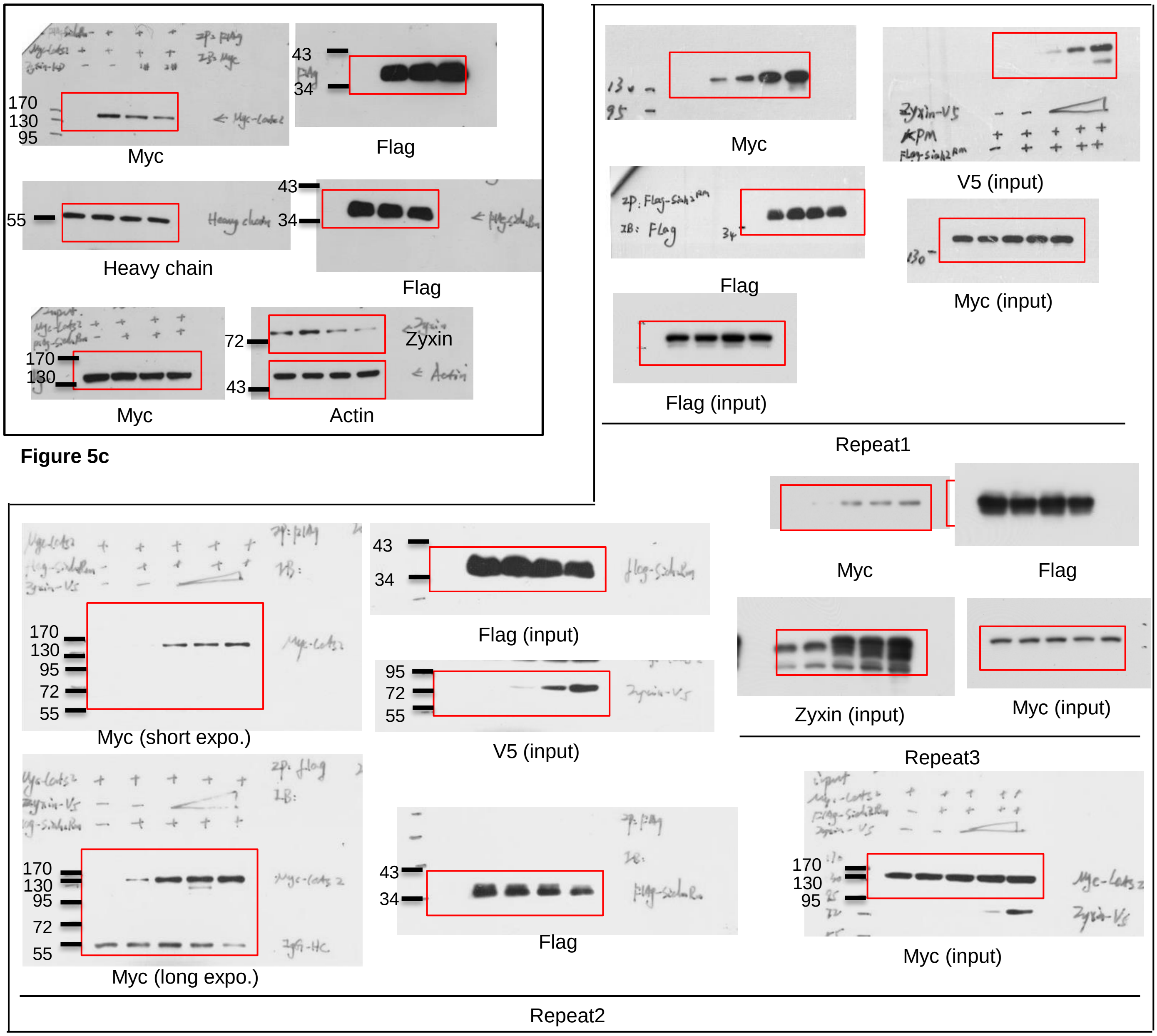

Figure 5c

Figure 5d

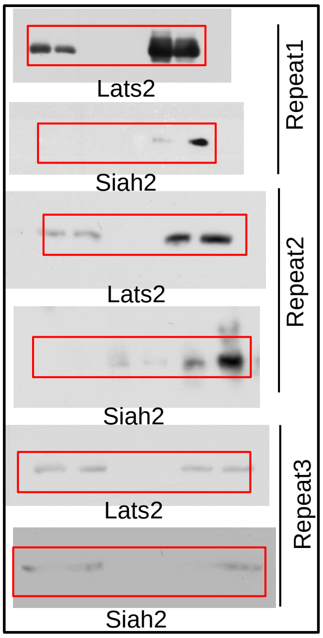

Figure 5f

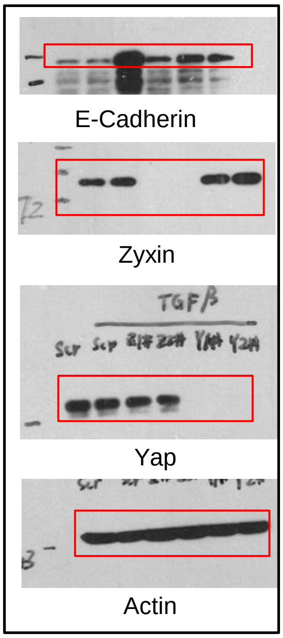

Figure 6b

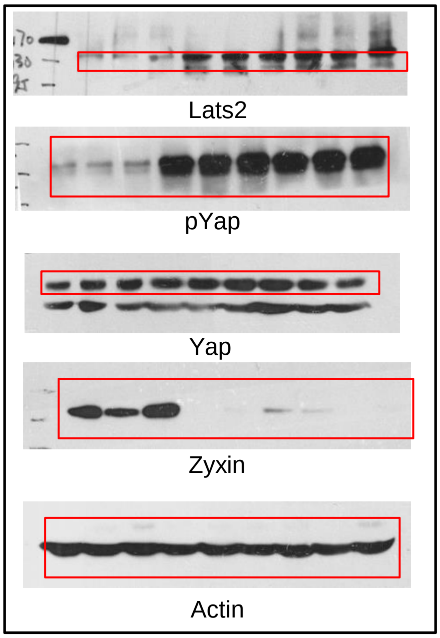

Figure 6i

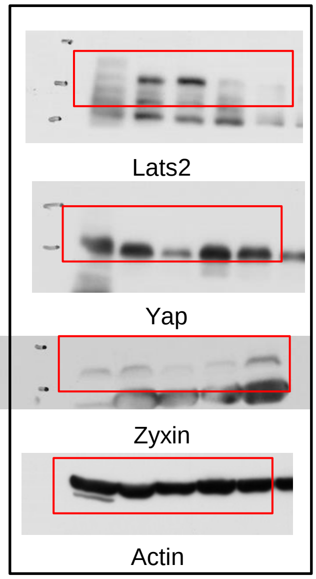

Figure 7a

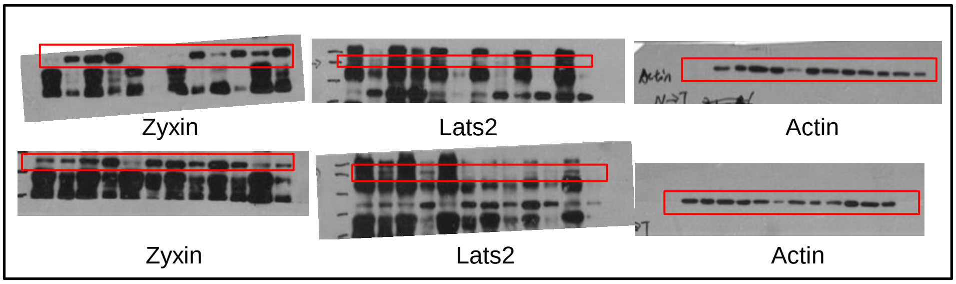

Figure 7b

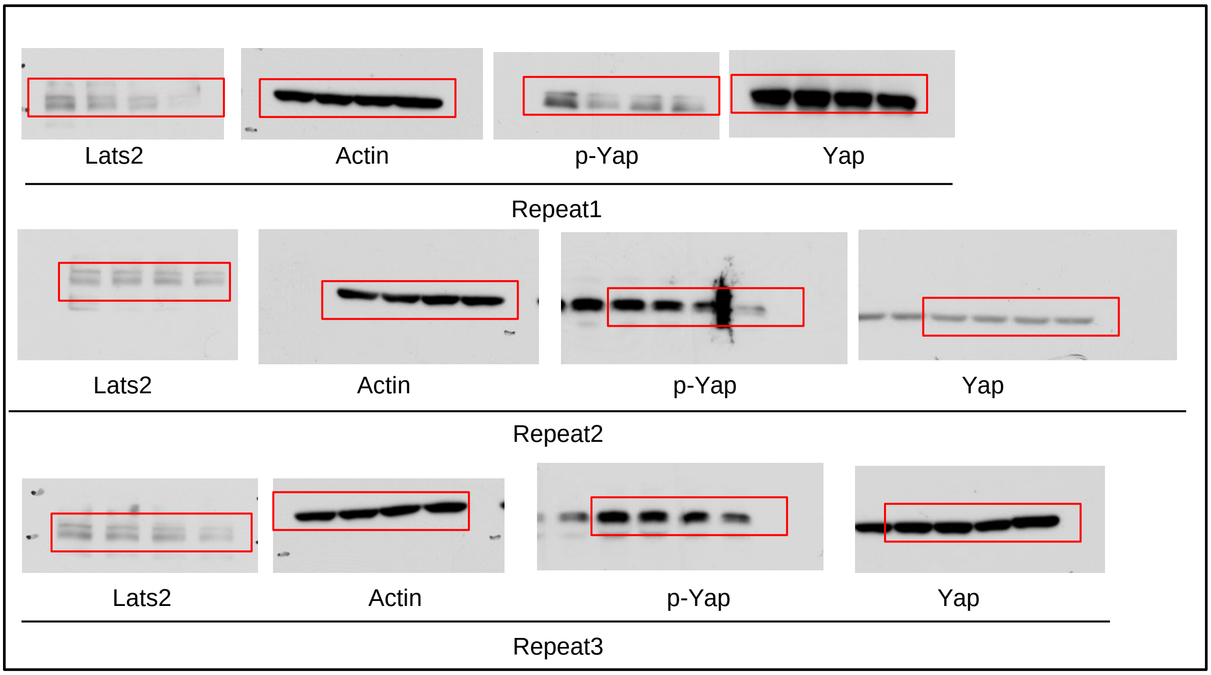

Supplementary Figure 1a

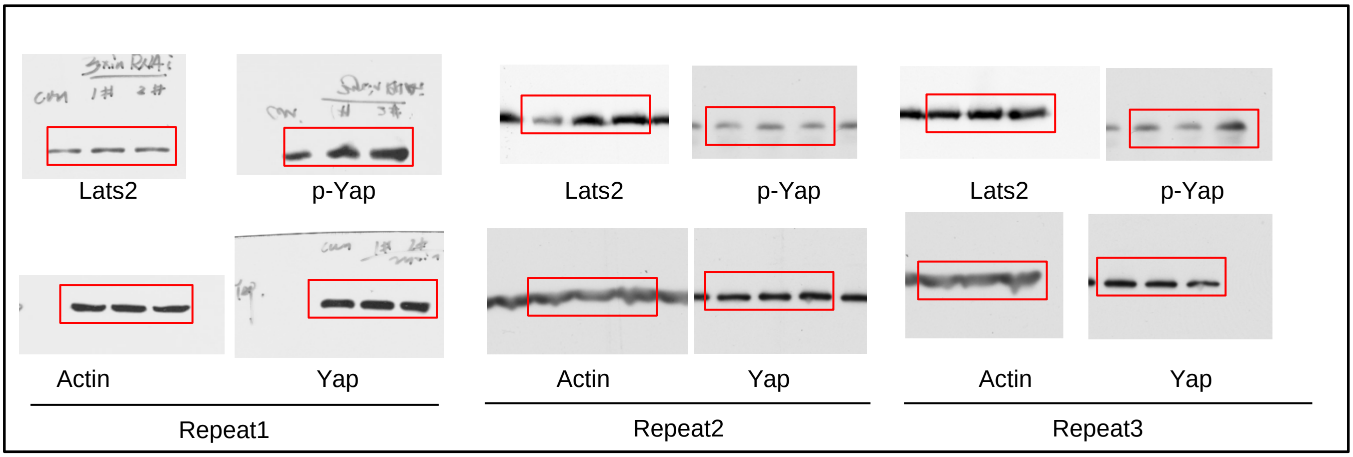

Supplementary Figure 1c

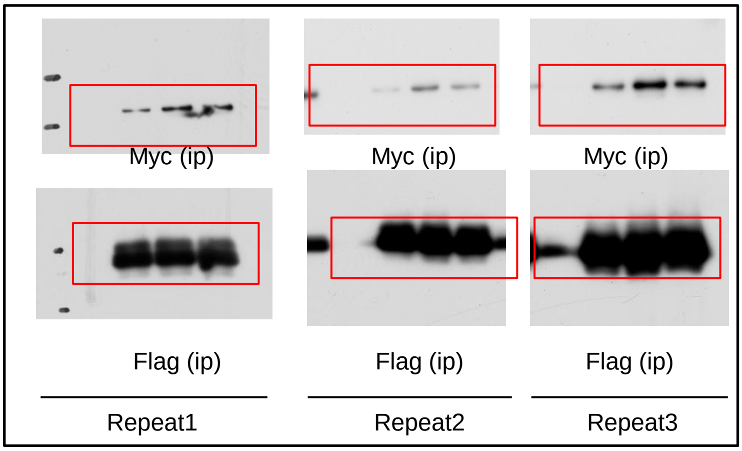

Supplementary Figure 3a

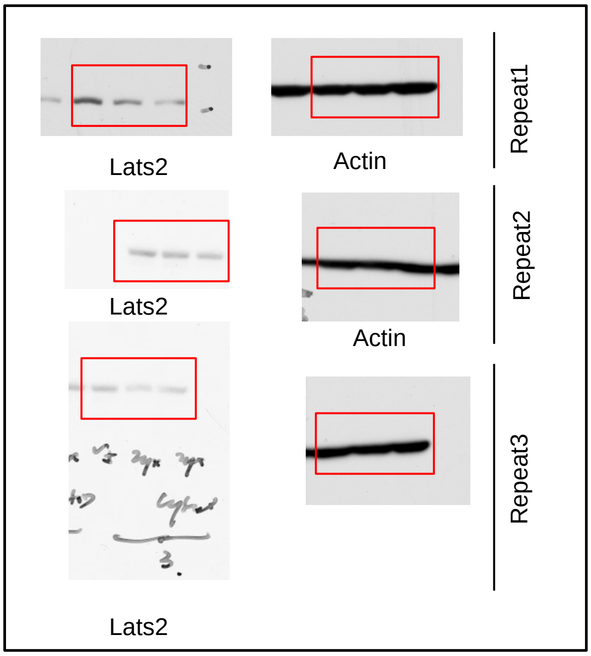

Supplementary Figure 3b

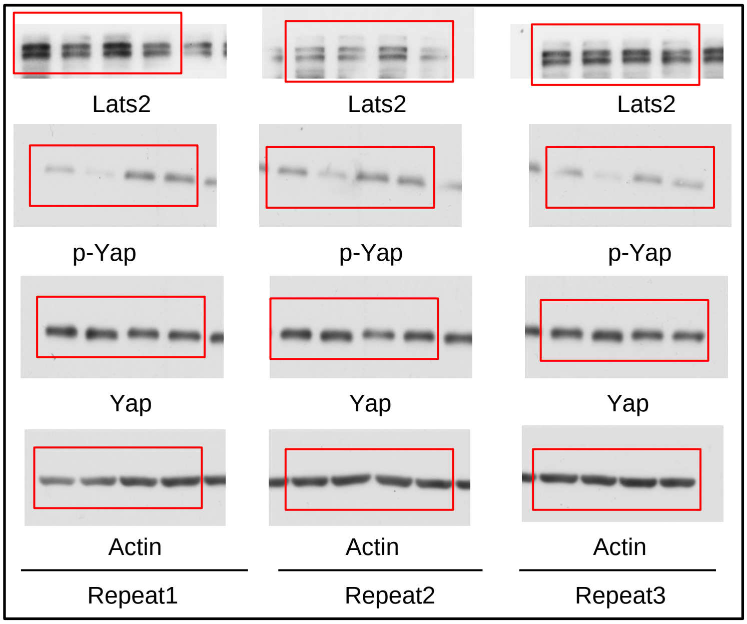

Supplementary Figure 3c
